# Incorporation of thio-pseudoisocytosine into triplex-forming peptide nucleic acids for enhanced recognition of RNA duplexes

**DOI:** 10.1093/nar/gkt1367

**Published:** 2014-01-13

**Authors:** Gitali Devi, Zhen Yuan, Yunpeng Lu, Yanli Zhao, Gang Chen

**Affiliations:** Division of Chemistry and Biological Chemistry, School of Physical and Mathematical Sciences, Nanyang Technological University, 21 Nanyang Link, Singapore 637371

## Abstract

Peptide nucleic acids (PNAs) have been developed for applications in biotechnology and therapeutics. There is great potential in the development of chemically modified PNAs or other triplex-forming ligands that selectively bind to RNA duplexes, but not single-stranded regions, at near-physiological conditions. Here, we report on a convenient synthesis route to a modified PNA monomer, thio-pseudoisocytosine (L), and binding studies of PNAs incorporating the monomer L. Thermal melting and gel electrophoresis studies reveal that L-incorporated 8-mer PNAs have superior affinity and specificity in recognizing the duplex region of a model RNA hairpin to form a pyrimidine motif major-groove RNA_2_–PNA triplex, without appreciable binding to single-stranded regions to form an RNA–PNA duplex or, via strand invasion, forming an RNA–PNA_2_ triplex at near-physiological buffer condition. In addition, an L-incorporated 8-mer PNA shows essentially no binding to single-stranded or double-stranded DNA. Furthermore, an L-modified 6-mer PNA, but not pseudoisocytosine (J) modified or unmodified PNA, binds to the HIV-1 programmed −1 ribosomal frameshift stimulatory RNA hairpin at near-physiological buffer conditions. The stabilization of an RNA_2_–PNA triplex by L modification is facilitated by enhanced van der Waals contacts, base stacking, hydrogen bonding and reduced dehydration energy. The destabilization of RNA–PNA and DNA–PNA duplexes by L modification is due to the steric clash and loss of two hydrogen bonds in a Watson–Crick-like G–L pair. An RNA_2_–PNA triplex is significantly more stable than a DNA_2_–PNA triplex, probably because the RNA duplex major groove provides geometry compatibility and favorable backbone–backbone interactions with PNA. Thus, L-modified triplex-forming PNAs may be utilized for sequence-specifically targeting duplex regions in RNAs for biological and therapeutic applications.

## INTRODUCTION

RNAs have an expanding list of biological functions including coding proteins, catalysis, gene regulation and immunomodulation (1–10). The functions of many RNAs are determined by the diverse structures they may form. RNA secondary structures are comprised of both single-stranded loop and double-stranded stem regions. RNA tertiary structures involve interactions between the secondary structure building blocks: for example, loop–stem, loop–loop and stem–stem interactions. RNA structures and functions are further diversified upon binding to proteins to form functional ribonucleoproteins such as snRNPs (11) and to other molecules and ions (as is the case in metabolite-sensing riboswitches) (1,7,8,10). Antisense therapeutics and microarray technologies (12–15) involve sequence-specific binding of oligonucleotides to single-stranded regions of target RNAs and have both had major impacts on biology and advancing RNA-based therapeutics. In contrast, there are no widely applied methods for targeting double-stranded regions of RNA; thus there is great potential for developing methods that can target double-stranded regions of RNA, which make up the majority of nucleotides in many functional RNAs (16,17).

**Figure 1.**
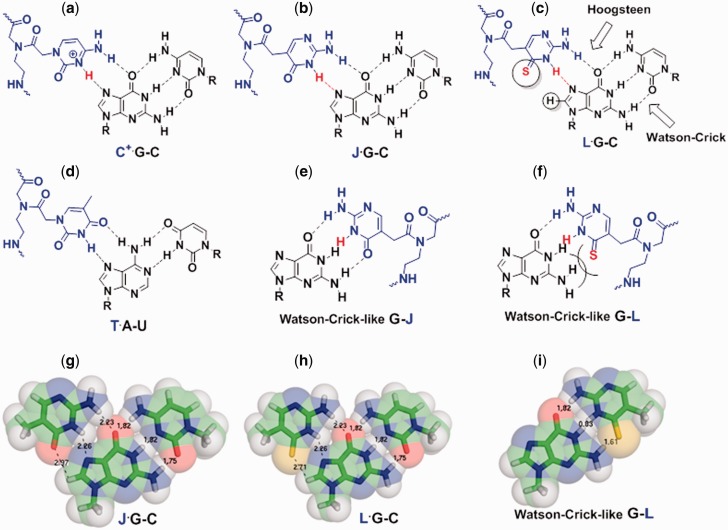
Chemical structures and structural models of base triples and base pairs formed between PNA (blue) and RNA (black). *H*^3^ and *S*^4^ atoms in bases C^+^, J and L are shown in red. (**a–d**) Chemical structures of base triples of C^+^∙G–C, J∙G–C, L∙G–C and T∙A–U. L has an enhanced van der Waals interaction with G in a Hoogsteen-like L∙G pair. (**e–f**) Chemical structures of Watson–Crick-like G–J and G–L pairs. (**g–i**) Structural models of base triples J∙G–C, L∙G–C and Watson–Crick-like G–L pair. The three dimensional coordinates are based on a C^+^∙G–C base triple from (26), assuming the structures do not change upon chemical modifications of base C. The numbers shown are inter-atomic distances in Å. van der Waals radii of N, O, S atom groups are ∼1.6, 1.5 and 1.8 Å, respectively (27). A steric clash occurs between G and L in a Watson–Crick-like G–L pair, if the base pairing interface of the G–L pair is maintained the same shape as that of a Watson–Crick G–C pair.

DNA and RNA duplexes are recognized by triplex-forming oligonucleotides (TFOs) through sequence-specific hydrogen bonding and base stacking interactions (18–26). Thus, TFOs have a great potential in biotechnology and therapeutics. However, formation of major groove C^+^∙G–C base triples is favored at relatively low pH (<6.0) due to the fact that cytosine *N*^3^ positions (p*K*_a_ = 4.5 for a C monomer) in TFOs need to be protonated to form stable C^+^∙G Hoogsteen pairs (Figure 1A). Numerous studies have been reported on enhancing DNA triplex stability at near neutral pH using chemically modified TFOs (18–20). For example, TFOs incorporating a neutral base pseudoisocytosine (J) (see Figure 1B for the nucleobase structure) show minimal pH dependence in DNA_2_–TFO triplex formation (19,28).

Peptide nucleic acids (PNAs), where the negatively charged sugar–phosphate backbone of natural nucleic acids is replaced by a neutral pseudopeptide backbone (see Figure 1 for the PNA backbone structure), show significantly enhanced (compared to unmodified DNA or RNA) binding affinity towards DNA duplexes (29–31). However, PNAs may also bind tightly to single-stranded DNAs (ssDNAs) in both parallel and anti-parallel orientations. Thus, strand invasion may occur, resulting in formation of DNA–PNA duplexes, DNA–PNA_2_ triplexes and other structures, instead of DNA_2_–PNA triplexes (29–37). PNAs incorporated with J monomers (Figure 1B and E) show minimal pH dependence in forming DNA–PNA_2_ triplexes (35).

A number of reports are focused on targeting RNA duplexes by RNA_2_–TFO triplex formation (21–24,38). Recently, RNA_2_–PNA triplex formation with minimal strand invasion was reported for unmodified and modified PNAs (39–43). It remains unclear whether it is possible to develop chemically modified PNAs or other triplex-forming ligands that bind to RNA duplex but not single-stranded regions at near-physiological condition.

A DNA TFO containing a ribonucleoside thio-pseudoisocytosine (see Figure 1C for the nucleobase structure) was found to enhance the formation of a DNA_2_–DNA triplex (44). We reason that thio-pseudoisocytosine has steric repulsion with G in a Watson–Crick-like pair (Figure 1F and I), but has enhanced van der Waals interactions with G in a Hoogsteen-like pair (Figure 1C and H). Thus, incorporation of thio-pseudoisocytosine monomers into PNAs may stabilize the RNA_2_–PNA triplex formation, destabilize the RNA–PNA duplex formation, and minimize the pH and salt dependence of RNA_2_–PNA triplex formation.

Here, we report on the synthesis and binding studies of PNAs incorporated with the modified PNA monomer, thio-pseudoisocytosine (L, Figure 1C and F). Thermal melting and gel electrophoresis studies were carried out to (i) determine the molecular determinants of and environment factors affecting parallel pyrimidine motif RNA_2_–PNA triplex stability, (ii) characterize the binding of PNAs to a DNA duplex and ssRNA and ssDNA and (iii) test the application of modified PNAs in targeting an HIV-1 ribosomal frameshift inducing RNA structure (45,46).

## MATERIALS AND METHODS

### General methods and synthesis of PNA monomer L

All anhydrous solvents were obtained from commercial sources. Reagents were used as received from commercial sources, without any further purification. Unless otherwise noted, commercial HPLC grade solvents and room temperature were used for all reactions. Thin layer chromatography (TLC) was performed to monitor reaction progress with aluminum sheets silica gel 60 F254 (Merck). Flash silica gel 230–400 mesh and ethyl acetate/petroleum ether mixture were used as eluting solvent for column chromatography. All ^1^H and ^13^C NMR spectra were acquired on a 300 MHz ^1^H (75 MHz, ^13^C) spectrometer. The mass of all compounds was characterized by high resolution mass spectrometry (electron ionization) [HRMS (EI)]. Chemically synthesized and reversed phase-high-performance liquid chromatography (RP-HPLC) purified RNA and DNA oligonucleotides were purchased from Sigma-Aldrich in Singapore. The detailed procedure for the synthesis of the modified PNA monomer L is shown in the Supplementary Material.

### Solid phase synthesis, purification and matrix-assisted laser desorption/ionization-time of flight analysis of PNA oligomers

The N-(2-aminoethyl)glycine PNA (*aeg*PNA) monomers were purchased from ASM Research Chemicals. PNA monomer J was synthesized following the reported method (35,47). Synthesis of PNA oligomers was carried out on 4-methylbenzhydrylamine hydrochloride (MBHA∙HCl) polystyrene based resin. The original loading value 1.5–1.7 mmol/g of this solid support was reduced to 0.35 mmol/g, using acetic anhydride as the capping reagent. (Benzotriazol-1-yloxy)tripyrrolidinophosphonium hexafluorophosphate (PyBop) and *N*,*N*-Diisopropylethylamine (DIPEA) were used as the coupling reagent and Boc strategy was followed during oligomer synthesis. After sequential deprotection of *t*-Boc group and coupling of *aeg*/modified PNA monomers on solid support, final cleavage of the oligomers were done by using ‘high-low trifluoroacetic acid (TFA)-trifluoromethanesulfonic acid (TFMSA)’ method. Oligomers were then precipitated with diethyl ether, dissolved in water and purified by RP-HPLC method using water-CH_3_CN-0.1% TFA as the mobile phase. Sample crystallization matrix α-cyano-4-hydroxycinnamic acid (CHCA) was used in matrix-assisted laser desorption/ionization-time of flight (MALDI-TOF) to characterize the oligomers.

### Thermal melting

UV absorbance versus temperature experiments were conducted using a Beckmann Coulter DU-800 spectrometer equipped with a Peltier temperature controller. Absorbance at 280 nm was recorded with increasing temperature from 15 to 95°C with a ramp rate of 0.5°C/min. The quartz cuvettes have an optical path length of 1 cm. Prior to each melting experiment, the RNA or DNA hairpin solution was heated at 95°C for 5 min and quickly cooled to ∼0°C (snap cooling). Subsequently, PNA/oligonucleotide was added to the snap cooled RNA or DNA hairpin solution and annealed by heating at 65°C for 5 min and slowly cooling to room temperature followed by incubation at 0°C for 3–5 h. The solution was covered with silicon oil to prevent evaporation during the melting experiment. The final concentration of each strand is 5 µM. *T*_m_’s were determined from the Gaussian fits of the first-derivative of the normalized curves. The buffers for studying salt concentration dependence contain 0.5 mM ethylenediaminetetraacetic acid (EDTA), 20 mM 4-(2-hydroxyethyl)-1-piperazineethanesulfonic acid (HEPES), pH 7.5 with varying [NaCl] (10, 100, 200, 300, 500 and 1000 mM). The buffers for studying pH dependence contain 200 mM NaCl, 0.5 mM EDTA, 20 mM 2-(*N*-morpholino)ethanesulfonic acid (MES) (pH 5.5 and 6.0) or HEPES (pH 6.5, 7.0, 7.5, 8.0, 8.5 and 9.0). We did not obtain equilibrium thermodynamic parameters from the thermal melting curves due to the hysteresis observed between heating and cooling curves. A ramp rate at 0.2°C/min did not reduce hysteresis significantly.

### Non-denaturing polyacrylamide gel electrophoresis

Non-denaturing polyacrylamide gel electrophoresis (PAGE) (12%) experiments were conducted with the sample incubation buffer containing 10 mM NaCl, 0.5 mM EDTA, 20 mM MES (pH 5.5), or 200 mM NaCl, 0.5 mM EDTA, 20 mM HEPES (pH 7.0, 7.5 and 8.0). The loading volume was 20 or 30 µl. Samples were prepared by snap cooling of the hairpin followed by annealing with PNAs/oligonucleotides by slow cooling from 65°C to room temperature followed by incubation at 4°C overnight. Thirty-five percent glycerol (20% of the total loaded volume) was added to the sample mixtures just before loading the samples into the wells. 1× TBE (Tris–Borate–EDTA) buffer, pH 8.3 was used as the running buffer for all experiments. The gel was run at 4°C at a voltage 250 V for 6 h. Gels were stained with ethidium bromide and imaged by a Typhoon phosphorimager.

## RESULTS AND DISCUSSION

### Synthesis of PNA monomer L and modified PNA oligomers

**Scheme 1.**
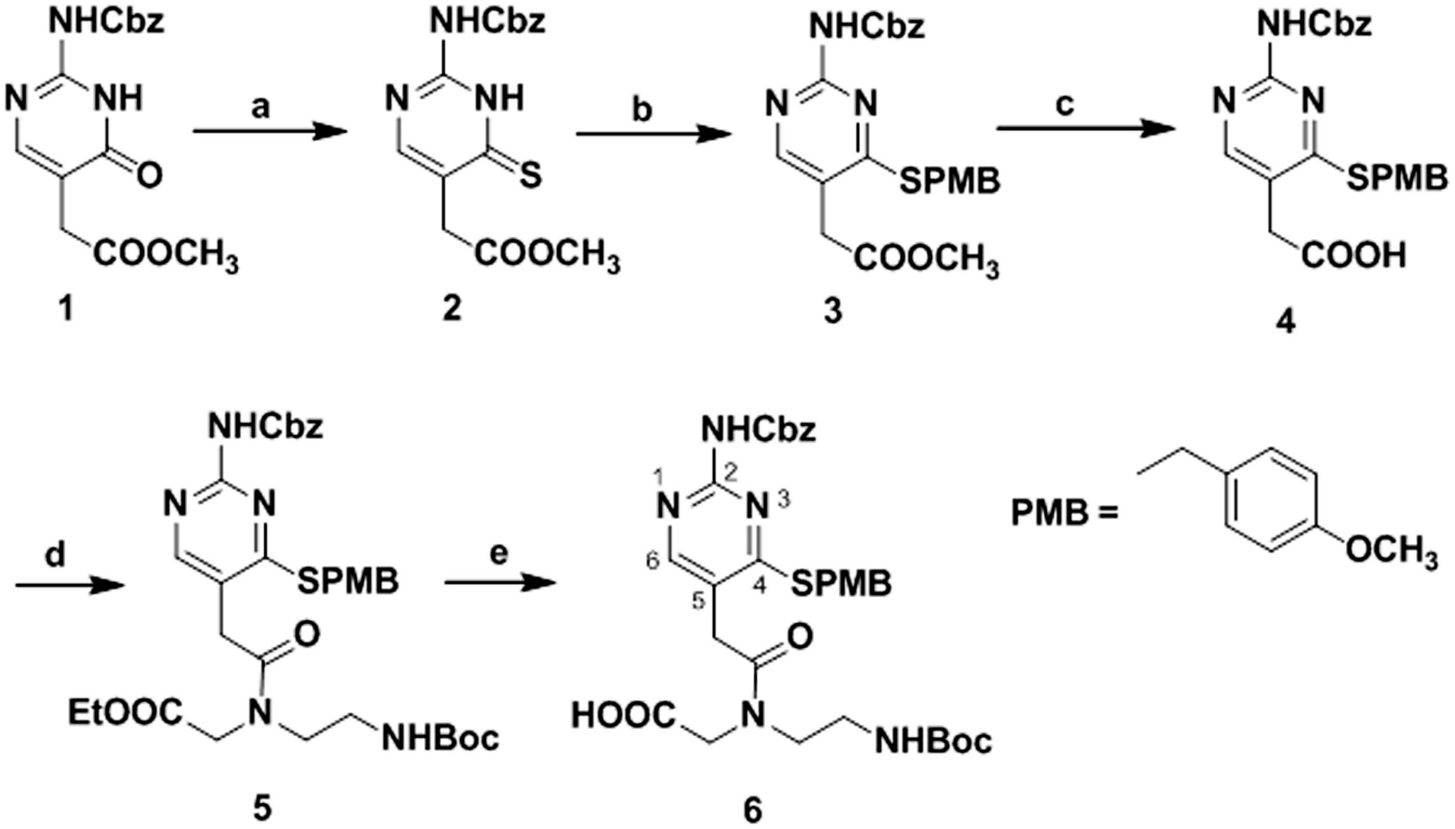
Reagents and conditions for PNA L monomer synthesis: (**A**) Lawesson’s reagent, THF, 0°C-rt, overnight, 54%. (**B**) 4-methoxybenzyl chloride, DCM, 1 h, 72%. (**C**) 1 M aqLiOH, THF, 1 h, 2 M HCl, 78%. (**D**) Ethyl *N*-(2-Boc-aminoethyl)glycinate, EDC∙HCl, DIPEA, DMF, 4 h, 0°C-rt, 58%. (**E**) 1 M aq LiOH, THF, 1 h, 70%.

A convenient route has been developed for the synthesis of PNA L monomer **6** protected at the *N^2^* exocyclic amine and *C^4^* thiocarbonyl group (Scheme 1). To synthesize methyl *N^2^*-(benzyloxycarbonyl)isocytosin-5-ylacetate **1**, we employed a previously reported method (35,47). The *C^4^* carbonyl of **1** was converted to its *C^4^* thiocarbonyl derivative **2** using Lawesson’s reagent (48–50). The sulfur group of the thiocarbonyl compound **2** was protected with 4-methoxybenzyl chloride in the presence of triethylamine to obtain the *N^2^* and *S* protected nucleobase methyl *N*^2^-(benzyloxycarbonyl)-*S^4^*-(4-methoxybenzyl)isocytosin-5-ylacetate **3**. The hydrolysis of **3** was accomplished using aqueous lithium hydroxide (LiOH), and a solid compound, *N*^2^-(benzyloxycarbonyl)-*S^4^*-(4-methoxybenzyl)isocytosin-5-ylacetic acid **4,** was obtained after acidification with 2 M hydrochloric acid. Compound **5** was obtained by coupling of **4** with the PNA backbone, i.e. ethyl *N*-(2-Boc-aminoethyl)glycinate (29,51) using 1-Ethyl-3-(3-dimethylaminopropyl)carbodiimide (EDC)/DIPEA as the coupling reagent. Further hydrolysis of **5** with aqueous LiOH followed by acidification with dowex cation exchange resin, yielded the final PNA monomer **6** in good yield. The protecting groups on *N^2^* exocyclic amine and *C^4^* thiocarbonyl group of monomer **6** were liberated under strong acidic conditions during the cleavage of PNA oligomers from resin after completion of solid phase synthesis. ^1^H and ^13^C NMR were used to characterize the compounds (Supplementary Figures S1–S6). A series of 8- and 6-mer PNAs with various modifications (Table 1, Supplementary Table S1 and Figure S7) were synthesized by solid phase peptide synthesis method. A lysine residue was incorporated at the N-terminus of the oligomers unless otherwise noted.

**Table 1.** Thermal stability comparison of triplexes (RNA_2_–PNA and DNA_2_–PNA) and duplexes (RNA–PNA and DNA–PNA)

PNA or oligonucleotide	Sequences^a^	Binding to rHP1 or dHP		Binding to ssRNA or ssDNA^b^	
		*T*_m1_	Δ*T*_m1_	*T*_m_	Δ*T*_m_
P8	LysNH-TCTCTTTC-CONH_2_	38.1 (<20)	NA	58.1 (37.7)	NA
J1-2	LysNH-TJTCTTTC-CONH_2_	38.1	0	52.3	−5.8
J1-4	LysNH-TCTJTTTC-CONH_2_	28.9	−9.2	52.1	−6.0
J1-8	LysNH-TCTCTTTJ-CONH_2_	25.5	−12.6	57.6	−0.5
J2-2,4	LysNH-TJTJTTTC-CONH_2_	33.5	−4.6	45.8	−12.3
J3	LysNH-TJTJTTTJ-CONH_2_	29.9 (<20)	−8.2	43.9 (37.7)	−14.2
L1-2	LysNH-TLTCTTTC-CONH_2_	45.0	+6.9	44.0	−14.1
L1-4	LysNH-TCTLTTTC-CONH_2_	43.7	+5.6	52.3	−5.8
L1-8	LysNH-TCTCTTTL-CONH_2_	36.0	−2.1	53.7	−4.4
L2-2,4	LysNH-TLTLTTTC-CONH_2_	55.6	+17.5	36.3	−21.8
clL2-2,4	H_2_N-TLTLTTTC-Lys-CONH_2_	54.0	+15.9	38.2	−19.9
L3	LysNH-TLTLTTTL-CONH_2_	64.1 (<20)	+26.0	27.8 (<20)	−30.3
apL3	LysNH-LTTTLTLT-CONH_2_	<20	NA	29.9	−28.2
R8	5′-UCUCUUUC-3′	<20	NA	33.0	−25.1
D8	5′-TCTCTTTC-3′	<20	NA	25.1	−33.0

**Figure 2.**
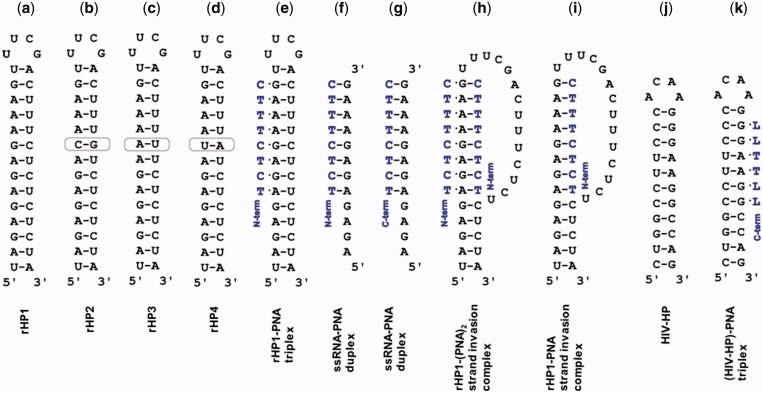
Structures studied in this article. Incorporation of L monomers into PNAs selectively stabilizes an RNA_2_–PNA triplex structure shown in panels (**e**) and (**k**), with minimal formation of alternative structures, e.g. those shown in panels (**f**–**i**). N-term: N-terminus. C-term: C-terminus. (**a–d**) Model RNA hairpins rHP1, rHP2, rHP3 and rHP4. (**e**) A model RNA_2_–PNA triplex formed between rHP1 and a PNA. (**f**) A parallel RNA–PNA duplex. (**g**) An anti-parallel RNA–PNA duplex. (**h** and **i**) Two possible strand invasion complexes rHP1–PNA_2_ and rHP1–PNA (52). (**j**) An HIV-1 programmed −1 ribosomal frameshift stimulatory RNA hairpin (HIV-HP). (**k**) A triplex formed between HIV-HP and PNA L4 (LLTTLL).

Buffer condition is 200 mM NaCl, 0.5 mM EDTA, 20 mM HEPES, pH 7.5. The concentration of each strand is 5 µM. All melting temperatures are shown in °C. ^a^All sequences have PNA backbone except that R8 and D8 have ribose-phosphate and deoxyribose–phosphate backbones, respectively. ^b^The sequence of the ssRNA and ssDNA is 5′˗AGAGAGAGAAAG˗3′ (Figure 2F and G), with ribose–phosphate and deoxyribose–phosphate backbones, respectively. The values shown in parentheses are for the binding to dHP or ssDNA. dHP is homologous to rHP1. PNA L3 shows the highest RNA_2_–PNA triplex-forming *T*_m1_ and lowest RNA–PNA duplex-forming *T*_m_. NA, Not applicable.

### L modification enhances RNA_2_–PNA triplex stability with reduced pH dependence

**Figure 3.**
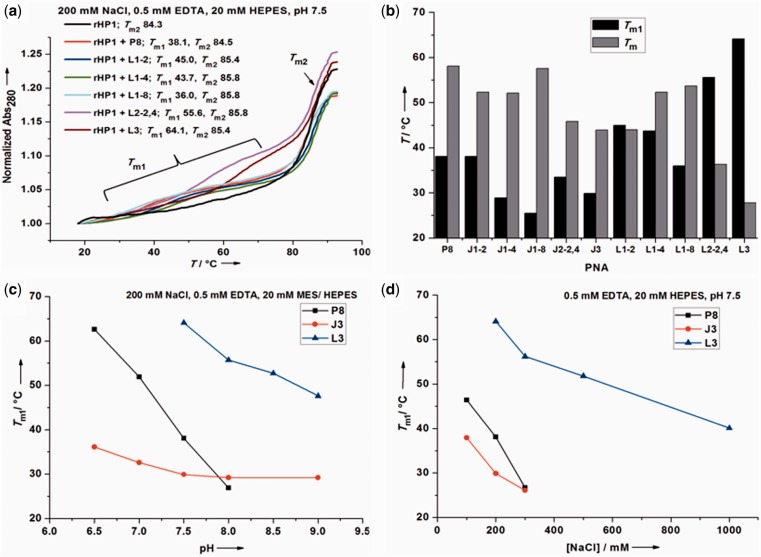
Thermal melting results for RNA_2_–PNA triplexes and RNA–PNA duplexes. (**a**) Normalized thermal melting curves for rHP1–PNA triplexes at 200 mM NaCl, 0.5 mM EDTA, 20 mM HEPES, pH 7.5. (**b**) Effects of J and L modifications on thermal stabilities of RNA_2_–PNA triplex (*T*_m1_) and RNA–PNA duplex (*T*_m_) at 200 mM NaCl, 0.5 mM EDTA, 20 mM HEPES, pH 7.5 (Table 1). PNA L3 shows the highest triplex-forming *T*_m1_ and lowest duplex-forming *T*_m_. (**c**) Effect of pH on thermal stabilities of rHP1–PNA triplexes (Table 2). (**d**) Effect of [NaCl] on thermal stabilities of rHP1–PNA triplexes (Table 3).

To test whether RNA_2_–PNA triplexes may form at near-physiological conditions, we first studied the binding of the 8-mer PNAs towards a model RNA hairpin (rHP1) (Figure 2A and E) (38) at various pHs and salt concentrations by UV absorbance detected thermal melting experiments. With increasing temperature, a PNA typically dissociates from rHP1 at *T*_m1_ before the melting of rHP1 at *T*_m2_ (*T*_m1_ ≤ *T*_m2_) (Figure 3A and Supplementary Figure S8).

For PNAs P8, J3 and L3, pH dependent thermal melting experiments were carried out at 200 mM NaCl. In the pH range studied (5.5–9.0), triplex *T*_m1_ decreases with increasing pH. Triplex *T*_m1_ for PNA L3 is always higher than PNAs J3 and P8 (Table 2 and Figure 3C). Remarkably, PNA L3 binds to rHP1 even at pH 9.0 with a *T*_m1_ of 47.6°C. The triplex melting transitions start to merge with hairpin rHP1 melting at pH 7.0 and 6.0, respectively, for PNAs L3 and P8. Triplex *T*_m1_ for unmodified PNA P8 shows the highest pH dependence (Figure 3C). These results are consistent with the fact that the apparent p*K*_a_ for *N*^3^ in a C nucleobase in a TFO of a DNA/RNA triplex is significantly shifted up to near neutral (9,53).

**Table 2.** pH dependent thermal melting results for rHP1–PNA triplexes and rHP1 alone

pH	rHP1–P8		rHP1–J3		rHP1–L3		rHP1
	*T*_m1_	*T*_m2_	*T*_m1_	*T*_m2_	*T*_m1_	*T*_m2_	*T*_m2_
5.5	(84.6)	84.6	NC	83.1	(82.9)	82.9	82.4
6.0	(84.5)	84.5	NC	84.1	(84.0)	84.0	83.1
6.5	62.6	83.8	36.1	84.6	(83.8)	83.8	84.2
7.0	51.9	84.6	32.6	84.6	(84.0)	84.0	83.5
7.5	38.1	83.3	29.9	84.8	64.1	83.9	84.3
8.0	26.9	84.6	29.2	83.8	55.7	83.9	83.6
8.5	<20.0	83.9	NC	84.1	52.7	84.6	83.6
9.0	<20.0	82.7	29.2	82.6	47.6	83.3	82.5

Buffers contain 200 mM NaCl, 0.5 mM EDTA, 20 mM MES (pH 5.5 and 6.0) or HEPES (pH 6.5–9.0). All melting temperatures are shown in °C. Values shown in parentheses are for melting traces with *T*_m1_ merging with *T*_m2_. NC, the melting is not clear due to a broad transition.

Previous research shows that the p*K*_a_ decrease toward neutral for *N*^3^ in 2-thio U (p*K*_a_ = 8.8) relative to U (p*K*_a_ = 9.3) monomer (Figure 1D) may enhance, respectively, the Watson–Crick (14,54) and Hoogsteen hydrogen bonding (38). Consistently, our quantum mechanics calculations have revealed that the gas phase local p*K*_a_ values for *N*^3^ atoms decrease upon thiolation (L versus J, 2-thio T versus T and 2-thio C versus C; Supplementary Tables S2 and S3). Thus, the p*K*_a_ decrease toward neutral for *N*^3^ in L monomer relative to J monomer (p*K*_a_ = 9.4) (19,35,55) may enhance the Hoogsteen hydrogen bonding (Figure 1B, C, G, and H) (56).

The stabilization effect of L modification may also result from the improved van der Waals contact between a sulfur atom in an L nucleobase in a triplex-forming PNA (TFPNA) and an *H*^8^ atom of a guanine in an RNA duplex (Figure 1C and H) (38,57,58). Furthermore, a nucleobase L (with a relatively more polarizable and less electronegative thio group) has enhanced stacking interactions with flanking nucleobases and has a reduced dehydration penalty (14,38,44,54,57–60).

### L modification reduces the salt dependence for RNA_2_–PNA triplex formation

We investigated salt concentration dependent RNA_2_–PNA triplex formation for PNAs P8, J3 and L3 by thermal melting at pH 7.5. Upon increasing [NaCl], the RNA hairpin is stabilized, whereas the triplex is destabilized (Table 3 and Figure 3D). The hairpin is stabilized with increasing [NaCl] because Na^+^ is condensed upon hairpin stem formation.

**Table 3.** [NaCl] dependent thermal melting results for rHP1–PNA triplexes and rHP1 alone

[NaCl] (mM)	rHP1–P8		rHP1–J3		rHP1–L3		rHP1
	*T*_m1_	*T*_m2_	*T*_m1_	*T*_m2_	*T*_m1_	*T*_m2_	*T*_m2_
10	(76.2)	76.2	(74.5)	74.5	(75.9)	75.9	74.1
100	46.4	81.6	37.9	82.2	(82.2)	82.2	81.5
200	38.1	84.6	29.9	83.5	64.1	85.4	84.3
300	26.7	87.4	26.1	85.2	56.2	87.4	87.1
500	<20	88.9	<20	89.6	51.8	89.3	88.6
1000	<20	>90	<20	>90	40.1	>90	>90

All buffers contain 0.5 mM EDTA, 20 mM HEPES, pH 7.5. All melting temperatures are shown in °C. Values shown in parentheses are for melting traces with *T*_m1_ merging with *T*_m2_.

Triplex structures with one or more C^+^∙G–C base triples and with all three strands comprising negatively charged DNA and/or RNA are destabilized upon increasing [NaCl] (25,38,61). This is likely due to the favorable charge–charge attraction between C^+^ and phosphate backbone decreases with increasing salt concentration, resulting in reduced triplex thermal stability. Similarly, PNA P8 forms less stable RNA_2_–PNA triplex with higher [NaCl], mainly due to the formation of three positively charged C^+^∙G–C base triples, which have favourable charge–charge attraction with negatively charged RNA backbone. In addition, all PNAs have one positively charged lysine residue (Table 1), which also has charge–charge attractions with RNA backbone. Furthermore, increasing [NaCl] may decrease the p*K*_a_ further away from neutral for *N^3^* in C residues (p*K*_a_ = 4.5 for C monomer) in PNA P8 (25,62). Thus, PNA binding to negatively charged RNA duplex is weakened upon increasing [NaCl]. PNA L3 and J3 show less pronounced [NaCl] dependent *T*_m1_ for triplex formation than PNA P8 (Table 3 and Figure 3D), presumably due to the absence of charged nucleobases in the RNA_2_–PNA triplexes containing L3 and J3. Taken together, RNA_2_–PNA triplex is significantly stabilized upon L modification in TFPNAs at near-physiological conditions with relatively small pH and salt concentration dependence.

### Effects of number and position of modifications on RNA_2_–PNA triplex formation

At 200 mM NaCl, pH 7.5, substitutions of a single C with an L monomer near to the N-terminus (L1-2) and middle (L1-4) positions of a PNA increase the *T*_m1_ by 6.9 and 5.6°C, respectively (Table 1 and Figure 3). For bi-modified (L2-2,4) and fully modified (L3) PNAs, *T*_m1_ values increase by 17.5 and 26.0°C, respectively. Triplex stability is relatively independent of the position of lysine (at N- or C-terminus) in a PNA (see L2-2,4 and clL2-2,4 in Table 1). The 8-mer oligonucleotides R8 and D8 do not form a stable triplex with rHP1 (Table 1), which shows that, compared to negatively charged RNA and DNA, PNAs have significantly enhanced affinity towards RNA duplex regions.

Surprisingly, at 200 mM NaCl, pH 7.5, C-terminus modification (L1-8) decreases *T*_m1_ slightly (by 2.1°C) (Table 1 and Figure 3). This observation may be explained by the fact that the terminal base triple of an RNA_2_–PNA triplex has a relatively more hydrophilic local environment than those of internal base triples. Thus, at the terminus of an RNA_2_–PNA triplex, with only one stacking partner, a neutral C∙G–C base triple or a positively charged C^+^∙G–C base triple is more favourable than a neutral and more hydrophobic L∙G–C base triple. The apparent p*K*_a_ of a terminal C nucleobase (apparent p*K*_a_ is ∼6–7) in a TFO of a DNA triplex is significantly lower than that of an internal one (with apparent p*K*_a_ > 8) (53). Thus, we expect the destabilization effect of a terminal L monomer to be more significant at low pH. The rHP1–L3 triplex, however, is more stable than rHP1–(L2-2,4) triplex with an additional C-terminal L∙G–C base triple. It is probably due to non-nearest neighbor (allosteric) effect caused by the two pre-existing L∙G–C base triples in the rHP1–(L2-2,4) triplex. The advantage of a neutral nucleobase L is that the triplex formation has low pH and salt dependence, as discussed above. In addition, the potential destabilizing effect due to charge–charge repulsion between adjacent positively charged C^+^∙G–C base triples (25,58) is not expected for neutral L∙G–C base triples.

At pH 8.0 or above, PNA J3 forms a more stable RNA_2_–PNA triplex than unmodified PNA P8 (Figure 3 and Tables 1–3), which is consistent with previous DNA–PNA_2_ and DNA_2_–TFO triplex studies (28,35). A destabilization effect of J modification, however, is observed at pH < 8, probably due to less favorable stacking interactions compared to C or C^+^. The destabilizing effect of J modification was observed at both internal and terminal positions. Thus, thiolation of J (to make L) is critical in stabilizing RNA_2_–PNA triplexes.

**Figure 4.**
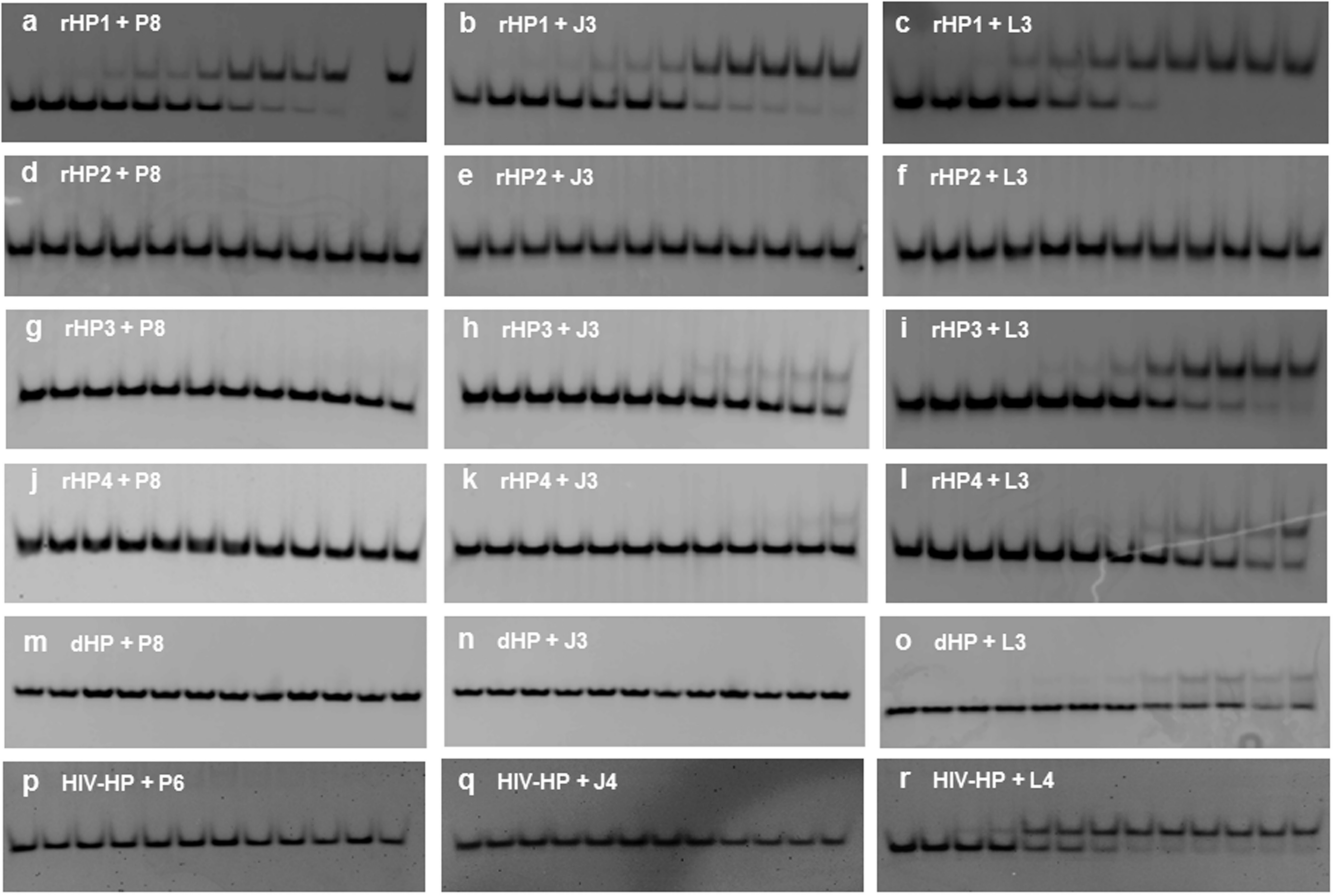
Non-denaturing PAGE (12%) with a running buffer of 1× TBE, pH 8.3 for 6 h at 250 V. The incubation buffer is 200 mM NaCl, 0.5 mM EDTA, 20 mM HEPES, pH 7.5. The loaded hairpins are at 1 µM in 20 µl. PNA concentrations in lanes from left to right are 0, 0.2, 0.4, 1, 1.6, 2, 4, 10, 16, 20, 28, 50 and 80 µM, respectively. (**a–c**) rHP1 binds to PNAs P8, J3 and L3 with *K*_d_ values of (5.3 ± 1.3), (7.0 ± 1.9) and (1.7 ± 0.6) µM, respectively. (**d–f**) rHP2 does not bind to PNAs P8, J3 or L3. (**g–i**) rHP3 shows no binding to P8 and weak binding to J3, and binds to L3 with *K*_d_ value of (12.2 ± 3.8) µM. (**j–l**) rHP4 shows no binding to P8, and weak binding to J3 and L3. (**m–o**) dHP is homologous to rHP1, and shows no binding to P8 or J3, and weak binding to L3. (**p–r**) Binding studies of PNAs P6 (CCTTCC), J4 (JJTTJJ) and L4 (LLTTLL) to HIV-HP. Only PNA L4 binds to HIV-HP, with a *K*_d_ value of (1.1 ± 0.3) µM.

We further studied RNA_2_–PNA triplex formation by non-denaturing PAGE. The fast- and slow-moving bands (Supplementary Figure S9, Figure 4) correspond to the hairpin and triplex, respectively. PNAs form parallel triplexes with the complementary rHP1, whereas control oligonucleotides R8 and D8 do not bind to rHP1 (Supplementary Figure S9, top panel), consistent with our thermal melting results. PNAs do not form triplexes with an RNA hairpin, rHP2 (Figure 2B), with one G–C pair inverted compared to rHP1 (Supplementary Figure S9, top panel).

We tested the binding of an anti-parallel PNA apL3 (Table 1) to rHP1. Our thermal melting results show no triplex melting transitions (at >20°C) for apL3 binding to rHP1 at pH 5.5–7.5 (Supplementary Figure S8C). However, at a relatively low temperature (4°C), two slow-moving bands are observed in non-denaturing PAGE (Supplementary Figure S9, bottom panel). Thus, PNA apL3 may form two different complexes with rHP1. We speculate that a 5-base-triple parallel triplex and two coaxially stacked 5-base-triple parallel triplexes may form between rHP1 and apL3 (Supplementary Figure S9, bottom panel). Interestingly, non-denaturing PAGE suggests that apL3 does not bind to rHP2, probably because one base triple and the coaxial stacking are disrupted (Supplementary Figure S9, bottom panel). Thus, coaxial stacking interactions may be utilized to enhance the formation of multiple RNA_2_–PNA triplexes on one RNA duplex.

### L modification destabilizes RNA–PNA and DNA–PNA duplexes

The relative stabilities of RNA–RNA (Figure 2A–D) and RNA–PNA (Figure 2F and G) duplexes determine the likelihood of strand invasion (Figure 2H and I). Modifications that stabilize Hoogsteen base pairs but destabilize Watson–Crick base pairs (Figure 1) enhance RNA_2_–PNA triplex formation, but minimize RNA–PNA duplex formation. We thus carried out thermal melting studies at 200 mM NaCl, pH 7.5 for PNAs binding to a ssRNA (Table 1) to form parallel and anti-parallel RNA–PNA duplexes (Figure 2F and G). A gradual decrease in RNA–PNA duplex *T*_m_ was observed with an increasing number of J and L modifications (Table 1 and Figure 3B). Remarkably, PNA L3 shows lowest RNA–PNA duplex *T*_m_ (27.8°C) [but highest RNA_2_–PNA triplex *T*_m1_ (64.1°C)]. In addition, *T*_m_ values are 37.7, 37.7 and <20°C, respectively, for PNAs P8, J3 and L3 binding to a homologous ssDNA (Table 1, Supplementary Figure S8Y and Z).

The destabilization effect of L modification on Watson–Crick-like RNA–PNA and DNA–PNA duplex formation is presumably due to the steric clash between the sulfur atom of L and the amino group of G in a Watson–Crick-like G–L base pair and loss of two hydrogen bonds (Figure 1F and I). Thus, incorporation of L monomers facilitates tight binding of PNAs to RNA duplexes, but not ssRNAs or ssDNAs.

### RNA_2_–PNA and DNA_2_–PNA triplex stability and strand invasion property measured by non-denaturing PAGE

Strand invasion is favored at relatively low salt concentration (29–35,52,63). Thus, we tested the possibility of strand invasion of PNAs at 10 mM NaCl, pH 5.5. As indicated by the non-denaturing PAGE results, PNA P8 shows strand invasion with rHP1, but PNA L3 forms RNA_2_–PNA triplexes without strand invasion (Supplementary Figure S10). Both triplex and hairpin rHP1 band intensities decrease with increasing concentration of PNA J3 (Supplementary Figure S10), indicating that aggregation may occur at this condition. As expected, PNAs P8 and J3 show strand invasion with a homologous DNA hairpin (dHP) of rHP1 (Supplementary Figures S11 and S12) at 10 mM NaCl, pH 5.5. Under the same conditions PNA L3 is still best for DNA_2_–PNA triplex formation without strand invasion. Thus, L modification minimizes strand invasion and stabilizes the formation of both RNA_2_–PNA and DNA_2_–PNA triplexes. Interestingly, when there is no appreciable strand invasion at 10 mM NaCl, pH 5.5, PNAs J3 and L3 bind more tightly to rHP1 than dHP (Supplementary Figures S10 and S12), consistent with previously reported results (41,43). It is likely that a relatively deep and narrow RNA duplex major groove provides geometry compatibility and favorable backbone–backbone interactions with PNA.

At 200 mM NaCl, PNAs P8, J3 and L3 do not show strand invasion of rHP1 with the PNA concentration up to ∼50 µM (Supplementary Figure S10A–C). Thus, we quantified the binding affinity by non-denaturing PAGE at 200 mM NaCl, pH 7.5 and 8.0. At pH 7.5, the dissociation constant (*K*_d_) values are (5.3 ± 1.3), (7.0 ± 1.9) and (1.7 ± 0.6) µM for rHP1 binding to PNAs P8, J3 and L3, respectively (Table 4, Figure 4A–C, Supplementary Figure S13). At 200 mM NaCl, pH 8.0, the *K*_d_ values are >20, (12.4 ± 4.2) and (3.8 ± 1.9) µM, respectively, for PNAs P8, J3 and L3 binding to rHP1 (Table 4, Supplementary Figures S14 and S15). The order of the binding affinities is consistent with our thermal melting results at both pH 7.5 and 8.0 (Figure 3 and Tables 1 and 2).

**Table 4.** *K*_d_ (µM) values for PNA binding to RNA or DNA duplexes obtained by non-denaturing PAGE

	P8	J3	L3
rHP1	5.3 ± 1.3 (>20)	7.0 ± 1.9 (12.4 ± 4.2)	1.7 ± 0.6 (3.8 ± 1.9)
rHP2	NB	NB	NB
rHP3	NB	>50	12.2 ± 3.8
rHP4	NB	>50	>50
dHP	NB	NB	>50

The incubation buffer contains 200 mM NaCl, 0.5 mM EDTA, 20 mM HEPES, pH 7.5. Values shown in parentheses are for *K*_d_’s measured at pH 8.0. NB, no binding.

Non-denaturing PAGE results show weak binding for PNA L3, and no binding for PNAs P8 and J3 to the DNA hairpin dHP at 200 mM NaCl, pH 7.5 (Figure 4M–O), which are consistent with the DNA-binding studies at 10 mM NaCl, pH 5.5 (as discussed above) and previous studies (41,43). We further quantified the sequence specificity of RNA duplex recognition by PNA at 200 mM NaCl, pH 7.5. Upon changing a G–C pair in rHP1 to C–G (rHP2), A–U (rHP3) or U–A (rHP4) (Figure 2B–D), we observed no appreciable binding for most of the PNAs (Figure 4D–L, Table 4). Surprisingly, PNA L3 binds to rHP3 [*K*_d_ = (12.2 ± 3.8) µM] at 200 mM NaCl, pH 7.5 (Table 4, Figure 4I, Supplementary Figure S13). It is probably because rHP3 has five consecutive A–U pairs, resulting in increased RNA-duplex flexibility and thus reduced sequence specificity for PNA binding. Further studies are needed to better understand the sequence specificity at varied sequence contexts. Taken together, our thermal melting and gel results suggest that L modified PNAs bind tightly and sequence-specifically to RNA-duplex regions, but not ssRNAs, ssDNAs or double-stranded DNAs.

### Binding of L-modified PNA to an HIV-1 programmed −1 ribosomal frameshift stimulatory RNA hairpin

We next studied the binding of 6-mer PNAs (Figure 4P–R, Supplementary Table S1) to an HIV-1 programmed −1 ribosomal frameshift stimulatory RNA hairpin (HIV-HP) (Figure 2J and K). In the secondary structure of HIV-1 RNA genome (64), the single-stranded 5′-GGAAGG-3′ sequence occurs frequently, however the duplex sequence 5′-GGAAGG-3′/3′-CCUUCC-5′ is not found outside of the ribosomal frameshift site. Remarkably, non-denaturing PAGE results (Figure 4R and Supplementary Figures S13–S15) reveal that the 6-mer L-modified PNA L4 (LLTTLL) binds to HIV-HP at 200 mM NaCl, pH 7.5 and 8.0, with *K*_d_ values of (1.1 ± 0.3) and (2.5 ± 0.6) µM, respectively. PNAs P6 and J4, however, show no binding at 200 mM NaCl, at pH 7.0 and 7.5 (Figure 4P and Q, and Supplementary Figure S16).

## CONCLUSION

In summary, we have developed a method for the synthesis of a novel PNA monomer thio-pseudoisocytosine (L). L-incorporated PNAs show superior affinity and specificity in recognizing RNA-duplex regions to form RNA_2_–PNA triplexes with minimal formation of RNA–PNA duplexes or RNA–PNA_2_ triplexes, at near-physiological conditions. In addition, L-modified short PNAs show no appreciable binding to ssDNA or double-stranded DNA. Triplex formation without strand invasion presumably has much faster kinetics than complex formation with strand invasion (52,63). The promising properties of L-modified PNAs suggest that carefully designed L-modified short PNAs can be used to specifically target double-stranded RNA structures and may thus be useful for mapping complex RNA secondary structures (by identifying double helices), probing RNA tertiary and RNA–protein interactions involving RNA duplex regions (by mapping double-stranded regions occluded by tertiary contacts or proteins), and specifically stabilizing desired RNA-duplex regions, which may prove useful in the application of modified PNAs as an RNA-targeting therapeutic (e.g. by stabilizing the HIV-1 frameshift hairpin).

## SUPPLEMENTARY DATA

Supplementary Data are available at NAR Online.

## FUNDING

Nanyang Technological University (NTU) (Start-up grant to G.C.); Singapore Ministry of Education Academic Research Fund Tier 1 (NTU internal grant to G.C.); the Singapore National Research Foundation Fellowship (NRF2009NRF-RF001-015 to Y.Z.). Funding for open access charge: NTU (Start-up grant to G.C.).

*Conflict of interest statement*. None declared.

## Supplementary Material

Supplementary Data
