# Supplementary material for: Incorporation of thio-pseudoisocytosine into triplex-forming peptide nucleic acids for enhanced recognition of RNA duplexes
Source: Nucleic Acids Res. 2014 Jan 13;42(6):4008–18. doi: 10.1093/nar/gkt1367 (PMC3973316; doi:10.1093/nar/gkt1367)
Supplement: Supplementary Data [file supp_42_6_4008__index.html]

Incorporation of thio-pseudoisocytosine into triplex-forming peptide nucleic acids for enhanced recognition of RNA duplexes — Incorporation of thio-pseudoisocytosine into triplex-forming peptide nucleic acids for enhanced recognition of RNA duplexes — Supplementary Data 

# Incorporation of thio-pseudoisocytosine into triplex-forming peptide nucleic acids for enhanced recognition of RNA duplexes

## Supplementary Data

files

**Files in this Data Supplement:**

- Supplementary Data - docx file
