# Supplementary material for: Incorporation of thio-pseudoisocytosine into triplex-forming peptide nucleic acids for enhanced recognition of RNA duplexes
Source: Nucleic Acids Res. 2014 Jan 13;42(6):4008–18. doi: 10.1093/nar/gkt1367 (PMC3973316; doi:10.1093/nar/gkt1367)
Supplement: Supplementary Data [file supp_gkt1367_nar-02304-f-2013-File007.docx]

**Supporting Information**

Incorporation of Thio-pseudoisocytosine into Triplex-Forming Peptide Nucleic Acids for Enhanced Recognition of RNA Duplexes

*Gitali Devi, Zhen Yuan, Yunpeng Lu, Yanli Zhao and Gang Chen*

Division of Chemistry and Biological Chemistry, School of Physical and Mathematical Sciences, Nanyang Technological University, 21 Nanyang Link, 637371 Singapore

**Table of Contents**

| 1. Synthesis of PNA monomer thio-pseudoisocytosine (L) | 2-5 |
| --- | --- |
| 1. 1H, 13C NMR spectra for synthesized compounds (Figures S1-S6) | 6-11 |
| 1. MALDI-TOF analysis of PNA oligomers (Table S1 and Figure S7) | 12-20 |
| 1. Thermal melting results (Figure S8) | 21-24 |
| 1. Non-denaturing PAGE results (Figures S9-S16) | 25-29 |
| 1. Computational details (Tables S2,S3) | 30 |
| 1. References | 31 |

1. **Synthesis of PNA monomer thio-pseudoisocytosine (L).**

*Methyl N^2^-(benzyloxycarbonyl)isocytosin-5-ylacetate* (**1**). To synthesize methyl *N^2^*-(benzyloxycarbonyl)isocytosin-5-ylacetate **1**, we employed a previously reported method (34,46). **^1^H NMR** (300 MHz, DMSO-d_6_): δ_H_ 3.35 (s, 2H, CH_2_COOCH_3_), 3.59 (s, 3H, COOCH_3_), 5.22 (s, 2H, PhCH_2_), 7.34-7.44 (m, 5H, PhCH_2_), 7.69 (s, 1H, H6), 11.51 (s, 2H, PhCH_2_OCONH, NHC4); **^13^C NMR** (75 MHz, CDCl_3_): δ_C_ 32.09 (CH_2_COOCH_3_), 52.14 (CH_2_COOCH_3_), 69.08 (PhCH_2_), 115.71 (C-5), 128.92-129.33 (PhCH_2_), 134.17 (PhCH_2_), 150.96 (C-6), 151.17 (C-2), 154.06 (PhCH_2_OCO), 160.33 (NHC4), 170.72 (CH_2_COOCH_3_); HRMS (EI): *m/z*: calculated for C_15_H_15_N_3_O_5_ (M + H)^+^ 318.1090, found: 318.1090.

*Methyl N*^2^*-(benzyloxycarbonyl)-C^4^-thioisocytosin-5-ylacetate (****2****).* Methyl *N*^2^-(benzyloxycarbonyl)isocytosin-5-ylacetate **1** (5.0 g, 15.7 mmol) was dissolved in anhydrous THF (70 mL) and cooled to 0 °C. Lawesson’s reagent (6.06 g, 15.7 mmol) was added slowly in small portions and the reaction mixture was allowed to reach room temperature gradually followed by stirring for overnight at rt. After completion of the reaction, compound was extracted with ethyl acetate (100 mL ×3). The combined organic layer was washed with saturated NaHCO_3_ followed by brine and dried over MgSO_4_. Purification by column chromatography yielded the product **2** (2.8 g, 54% yield) as a pale yellow solid. **^1^H NMR** (300 MHz, DMSO-d_6_): δ_H_ 3.59 (s, 3H, CH_2_COOCH_3_), 3.64 (s, 2H, CH_2_COOCH_3_), 5.28 (s, 2H, PhCH_2_), 7.36-7.46 (m, 5H, PhCH_2_), 7.93 (s, 1H, H6), 11.83 (br s, 1H, PhCH_2_OCONH), 12.81 (br s, 1H, NHC4); **^13^C NMR** (75 MHz, CDCl_3_): δ_C_ 36.33 (CH_2_COOCH_3_), 52.37 (CH_2_COOCH_3_), 69.71 (PhCH_2_), 127.17 (C-5), 129.21-129.59 (PhCH_2_), 134.10 (PhCH_2_), 148.35 (C-6), 150.09 (C-2), 153.91 (PhCH_2_OCO), 170.56 (CH_2_COOCH_3_), 183.60 (NHC4); HRMS (EI): *m/z*: calculated for C_15_H_15_N_3_O_4_S (M + H)^+^ 334.0862, found:334.0858.

*Methyl N^2^-(benzyloxycarbonyl)-S^4^-(4-methoxybenzyl)isocytosin-5-ylacetate* (**3**). 4-methoxybenzyl chloride (3.3 g, 23.11 mmol) was added slowly to a mixture of Methyl *N^2^*-(benzyloxycarbonyl)-*C^4^*-thioisocytosin-5-ylacetate **2** (7.0 g, 21.01 mmol) and triethylamine (4.2 g, 42.02 mmol) in anhydrous DCM (100 mL). After 1h, DCM (100 mL) was added to the reaction mixture. The organic layer was washed with water (75 mL ×1), saturated NaHCO_3_ (75 mL ×1) followed by brine (75 mL ×2) and dried over MgSO_4_. DCM was removed in rotavapour under reduced pressure. Column purification gives the compound **3** (6.6 g, 72% yield). **^1^H NMR** (300 MHz, DMSO-d_6_): δ_H_ 3.60 (s, 5H, CH_2_COOCH_3_, CH_2_COOCH_3_), 3.71 (s, 3H, OCH_3_), 4.48 (s, 2H, CH_2_S), 5.21 (s, 2H, PhCH_2_O), 6.80-6.83 (d, 2H, PhCH_2_S), 7.32-7.47 (m, 7H, PhCH_2_O, PhCH_2_S), 8.22 (s, 1H, H6), 10.60 (s, 1H, PhCH_2_OCONH); **^13^C NMR** (75 MHz, CDCl_3_): δ_C_ 33.58 (CH_2_S), 34.39 (CH_2_COOCH_3_), 52.27 (CH_2_COOCH_3_), 55.25 (OCH_3_), 67.47 (PhCH_2_O), 113.95 (PhCH_2_S) 119.02 (C-5), 128.57-128.98 (PhCH_2_O), 130.52 (PhCH_2_S), 135.75 (PhCH_2_S), 151.70 (PhCH_2_O), 155.65 (PhCH_2_OCO), 155.91 (C-2) 158.89 (C-6), 169.86 (C-4), 170.83 (CH_2_COOCH_3_); HRMS (EI): *m/z*: calculated for C_23_H_23_N_3_O_5_S (M + H)^+^ 454.1437, found: 454.1437.

*N^2^-(benzyloxycarbonyl)-S^4^-(4-methoxybenzyl)isocytosin-5-ylacetic acid* (**4**). Methyl *N^2^*-(benzyloxycarbonyl)*-S^4^-*(4-methoxybenzyl)isocytosin-5-ylacetate **3** (2.0 g, 8.83 mmol) was suspended in THF (20 mL) and cooled to 0 °C. 1 M lithium hydroxide (25 mL, 25 mmol) was added and the reaction mixture was stirred at 0 °C for 1h. After monitoring the reaction progress by TLC, the product was precipitated by acidifying to pH 2 with 1 M hydrochloric acid. Product **4** was collected by filtration, washed once with water and dried to give a white solid (1.5 g, 78% yield). **^1^H NMR** (300 MHz, DMSO-d_6_): δ_H_ 3.46 (s, 2H, CH_2_COOH), 3.69 (s, 3H, OCH_3_), 4.45 (s, 2H, PhCH_2_S), 5.26 (s, 2H, PhCH_2_O), 6.78-6.80 (d, PhCH_2_S), 7.33-7.42 (m, 7H, PhCH_2_O, PhCH_2_S), 8.17 (s, 1H, H6), 10.55 (s, 1H, PhCH_2_OCONH), 12.47 (bs, COOH); **^13^C NMR** (75 MHz, DMSO-d_6_): δ_C_ 32.36 (CH_2_COOH), 34.56 (PhCH_2_S), 55.48 (OCH_3_), 66.34 (PhCH_2_O), 114.24 (PhCH_2_S), 120.69 (C-5), 128.41-128.90 (PhCH_2_O), 130.28 (PhCH_2_S), 130.88 (PhCH_2_S), 136.98 (PhCH_2_S), 152.34 (PhCH_2_O), 156.12 (PhCH_2_OCO), 157.30 (C-2), 158.83 (C-6), 169.02 (C-4) 171.45 (COOH); HRMS (EI): *m/z*: calculated for C_22_H_21_N_3_O_5_S (M + H)^+^ 440.1280, found: 440.1278.

*Ethyl N-(2-Boc-aminoethyl)N-(N^2^-(benzyloxycarbonyl)-S^4^-(4-methoxybenzyl)isocytosin-5-ylacetate* (**5**). A mixture of methyl *N^2^*-(benzyloxycarbonyl)-*S^4^*-(4-methoxybenzyl)isocytosin-5-ylacetic acid **4** (2.0 g, 4.55 mmol), EDC hydrochloride (1.1 g, 5.46 mmol), DIPEA (1.76 g, 13.65 mmol) was dissolved in anhydrous DMF and was stirred at 0 °C for 10 mins under nitrogen atmosphere. Ethyl *N*-(2-Boc-aminoethyl)glycinate (1.1 g, 4.55 mmol) dissolved in minimum volume of DMF was added to the above cooled reaction mixture. Stirring was continued at rt for another 4 h followed by addition of dichloromethane (100 mL). The organic phase was washed with half-saturated aq NaHCO_3_ (30 mL ×2), half-saturated aq KHSO_4_ (30 mL ×2), brine (40 mL ×1) and dried over MgSO_4_. Organic layer was then evaporated under reduced pressure and compound **5** (1.7 g, 58% yield) was obtained upon column chromatography. **^1^H NMR** (300 MHz, CDCl_3_): δ_H_ 1.19-1.27 (t, 3H, CH_3_CH_2_), 1.39-1.41 (d, 9H, Boc), 3.24-3.28 (m, 2H, CH_2_NH), 3.43-3.51 (m, 4H, NCH_2_CH_2_, CH_2_CON), 3.75 (s, 3H, OCH_3_), 3.98 and 4.09 (d, 2H, ma and mi, respectively, COCH_2_N), 4.11-4.20 (q, 2H, COOCH_2_CH_3_), 4.46 (s, PhCH_2_S), 5.45 (s, 2H, PhCH_2_O), 6.76-6.79 (d, 2H, PhCH_2_S), 7.26-7.41 (m, 7H, PhCH_2_O, PhCH_2_S), 7.99 and 8.01 (d, 1H, mi and ma, respectively, H6), 9.50 (br s, NHBoc, 1H), 9.82 (br s, 1H, PhCH_2_OCONH); **^13^C NMR** (75 MHz, CDCl_3_): δ_C_ 14.07 (CH_2_CH_3_), 28.35 (Boc), 32.89 (CH_2_S), 33.30 and 33.55 (mi and ma, respectively, CH_2_CON), 38.87 (CH_2_NH), 49.04 (NCH_2_CH_2_), 49.75 (COCH_2_N), 55.20 (OCH_3_), 61.44 and 61.94 (ma and mi respectively, CH_3_CH_2_), 67.21 (PhCH_2_O), 79.26 and 79.68 (mi and ma respectively, OC(CH_3_)_3_), 113.68 (PhCH_2_S), 119.78 and 119.99 (mi and ma respectively, C-5), 128.33-128.72 (m, PhCH_2_O), 130.48 (PhCH_2_S), 135.86 (PhCH_2_S), 151.67 (PhCH_2_O), 155.37 and 155.45 (ma and mi, respectively, Z-CO), 155.98 (m, C-2, NHCOOC(CH_3_)_3_), 158.80 (C-6), 169.10-170.21 (m, C-4, CH_2_CON, CH_3_CH_2_OCO); HRMS (EI): *m/z*: calculated for C_33_H_41_N_5_O_8_S (M + H)^+^ 668.2754, found: 668.2756.

*N-(2-Boc-aminoethyl)N-(N^2^-(benzyloxycarbonyl)-S^4^-(4-methoxybenzyl)isocytosin-5-ylacetic acid* (**6**). *Ethyl N-(2-Boc-aminoethyl)N-(N^2^-(benzyloxycarbonyl)-S^4^-(4-methoxybenzyl)isocytosin-5-ylacetate* **5** (2.0 g, 2.99 mmol) was dissolved in THF (10 mL) and cooled to 0 °C. 1 M lithium hydroxide was added and reaction mixture was stirred at 0 °C for 1h. The product **6** was obtained as white solid (1.34 g, 70% yield) from the reaction mixture by acidifying to pH 2 with Dowex H^+^ resin. **^1^H NMR** (300 MHz, DMSO-d_6_): δ_H_ 1.34-1.36 (d, 9H, Boc), 3.01-3.61 (m, 6H, CH_2_NH, NCH_2_CH_2_, CH_2_CON), 3.71 (s, 3H, OCH_3_), 3.95 and 4.23 (d, 2H, ma and mi, respectively, COCH_2_N), 4.45 (s, 2H, PhCH_2_S), 5.21 (s, 2H, PhCH_2_O), 6.80-6.82 (d, 2H, PhCH_2_S), 7.28-7.42 (m, 7H, PhCH_2_O, PhCH_2_S), 8.06 and 8.07 (d, mi and ma, respectively, 1H, H6), 10.62 (s, 1H, PhCH_2_OCONH); HRMS (EI): *m/z*: calculated for C_31_H_37_N_5_O_8_S (M + H)^+^ 640.2441, found: 640.2443.

1. **^1^H, ^13^C NMR spectra for synthesized compounds.**

**Figure S1a**

**Figure S1b**

**Figure S2a**

**Figure S2b**

**Figure S3a**

**Figure S3b**

**Figure S4a**

**Figure S4b**

**Figure S5a**

**Figure S5b**

**Figure S6**

1. **MALDI-TOF analysis of PNA oligomers.**

**Table S1.** MALDI-TOF data for synthesized PNAs.

| PNA | Sequence | Molecular Formula | Calculated MW | Observed MW |
| --- | --- | --- | --- | --- |
| PNAs targeting rHP1 | | | | |
| P8 | LysNH-TCTCTTTC-CONH_2_ | C_91_H_124_N_38_O_30_ | 2230.18 | 2230.13 |
| J1-2 | LysNH-TJTCTTTC-CONH_2_ | C_91_H_124_N_38_O_30_ | 2230.18 | 2229.76 |
| J1-4 | LysNH-TCTJTTTC-CONH_2_ | C_91_H_124_N_38_O_30_ | 2230.18 | 2229.78 |
| J1-8 | LysNH-TCTCTTTJ-CONH_2_ | C_91_H_124_N_38_O_30_ | 2230.18 | 2229.96 |
| J2-2,4 | LysNH-TJTJTTTC-CONH_2_ | C_91_H_124_N_38_O_30_ | 2230.18 | 2230.10 |
| J3 | LysNH-TJTJTTTJ-CONH_2_ | C_91_H_124_N_38_O_30_ | 2230.18 | 2229.92 |
| L1-2 | LysNH-TLTCTTTC-CONH_2_ | C_91_H_124_N_38_O_29_S | 2246.26 | 2245.94 |
| L1-4 | LysNH-TCTLTTTC-CONH_2_ | C_91_H_124_N_38_O_29_S | 2246.26 | 2246.21 |
| L1-8 | LysNH-TCTCTTTL-CONH_2_ | C_91_H_124_N_38_O_29_S | 2246.26 | 2245.95 |
| L2-2,4 | LysNH-TLTLTTTC-CONH_2_ | C_91_H_124_N_38_O_28_S_2_ | 2262.33 | 2261.61 |
| clL2-2,4 | NH_2_-TLTLTTTC-Lys-CONH_2_ | C_91_H_124_N_38_O_28_S_2_ | 2262.33 | 2262.00 |
| L3 | LysNH-TLTLTTTL-CONH_2_ | C_91_H_124_N_38_O_27_S_3_ | 2278.33 | 2277.97 |
| apL3 | LysNH-LTTTLTLT-CONH_2_ | C_91_H_124_N_38_O_27_S_3_ | 2278.33 | 2300.17 [M + Na^+^] |
| PNAs targeting HIV-HP | | | | |
| P6 | LysNH-CCTTCC-CONH_2_ | C_68_H_95_N_31_O_21_ | 1682.67 | 1682.79 |
| J4 | LysNH-JJTTJJ-CONH_2_ | C_68_H_95_N_31_O_21_ | 1682.67 | 1682.70 |
| L4 | LysNH-LLTTLL-CONH_2_ | C_68_H_95_N_31_O_17_S_4_ | 1746.93 | 1746.60 |

|   **P8 (C_91_H_124_N_38_O_30_)**  **Calcd MW 2230.18**  **Obs MW 2230.13**  **Figure S7a** |
| --- |
|   **J1-2 (C_91_H_124_N_38_O_30_)**  **Calcd MW 2230.18**  **Obs MW 2229.76**  **Figure S7b** |
|   **J1-4 (C_91_H_124_N_38_O_30_)**  **Calcd MW 2230.18**  **Obs MW 2229.78**  **Figure S7c** |
|   **J1-8 (C_91_H_124_N_38_O_30_)**  **Calcd MW 2230.18**  **Obs MW 2229.96**  **[M + Na^+^]**  **Figure S7d** |
|   **J2-2,4 (C_91_H_124_N_38_O_30_)**  **Calcd MW 2230.18**  **Obs MW 2230.10**  **Figure S7e** |
|   **J3 (C_91_H_124_N_38_O_30_)**  **Calcd MW 2230.18**  **Obs MW 2229.92**  **Figure S7f** |
|   **L1-2 (C_91_H_124_N_38_O_29_S)**  **Calcd MW 2246.26**  **Obs MW 2245.94**  **Figure S7g** |
|   **L1-4 (C_91_H_124_N_38_O_29_S)**  **Calcd MW 2246.26**  **Obs MW 2246.21**  **Figure S7h** |
|   **L1-8 (C_91_H_124_N_38_O_29_S)**  **Calcd MW 2246.26**  **Obs MW 2245.95**  **Figure S7i** |
|   **L2-2,4 (C_91_H_124_N_38_O_28_S_2_)**  **Calcd MW 2262.33**  **Obs MW 2261.61**  **Figure S7j** |
|  |
|   **L3 (C_91_H_124_N_38_O_27_S_3_)**  **Calcd MW 2278.33**  **Obs MW 2277.97**  **Figure S7k** |
|   **apL3 (C_91_H_124_N_38_O_27_S_3_)**  **Calcd MW 2278.33**  **Obs MW 2300.17 [M + Na^+^]**  **Figure S7l** |
|   **clL2-2,4 (C_91_H_124_N_38_O_28_S_2_)**  **Calcd MW 2262.33**  **Obs MW 2262.00**  **Figure S7m** |

**P6 (C_68_H_95_N_31_O_21_) Calcd MW 1682.67 Obs MW 1682.79**

**Figure S7n**

|  **J4 (C_68_H_95_N_31_O_21_) Calcd MW 1682.67 Obs MW 1682.70**    **Figure S7o**    **J4 (C_68_H_95_N_31_O_21_) Calcd MW 1682.67 Obs MW 1682.70**    **Figure S7o**    **L4 (C_68_H_95_N_31_O_17_S_4_) Calcd MW 1746.93 Obs MW 1746.60**        **Figure S7p**     1. **Thermal melting results.**  \| 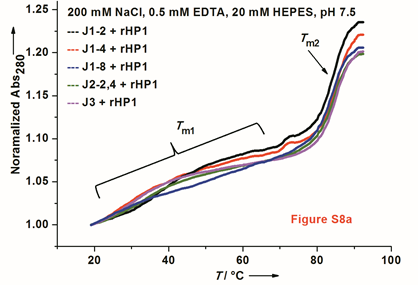 \| 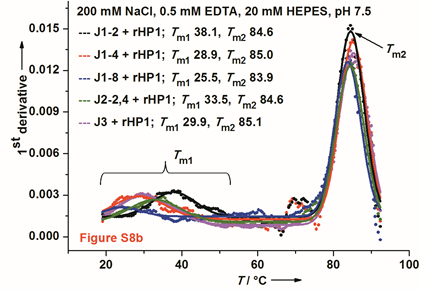 \| \| --- \| --- \| \| 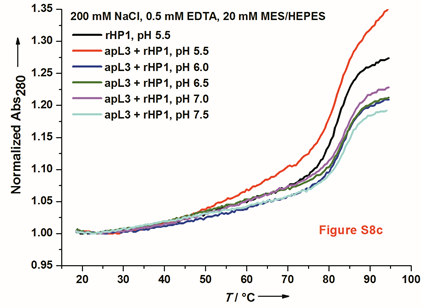 \| 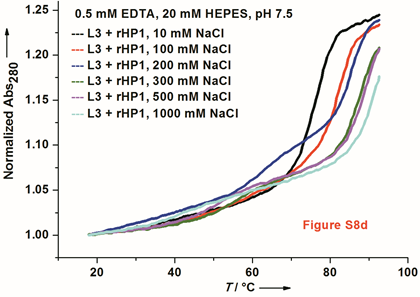 \| \| 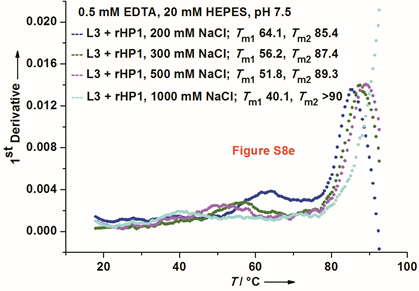 \| 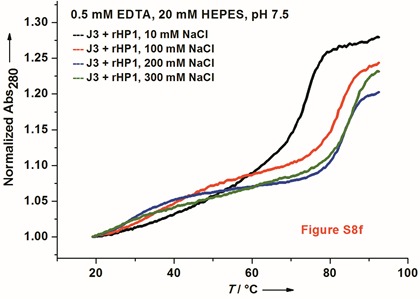 \| \| 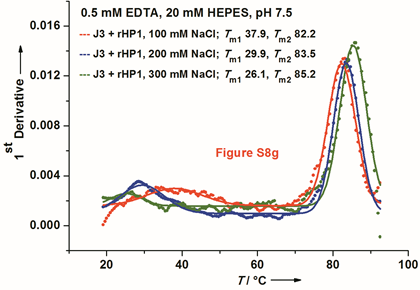 \| 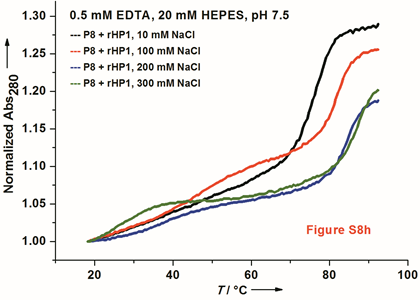 \| \| 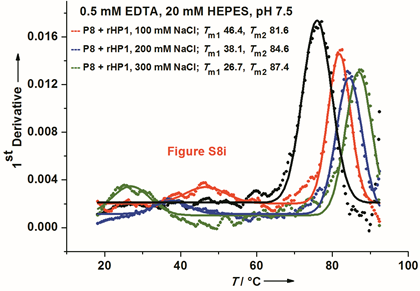 \| 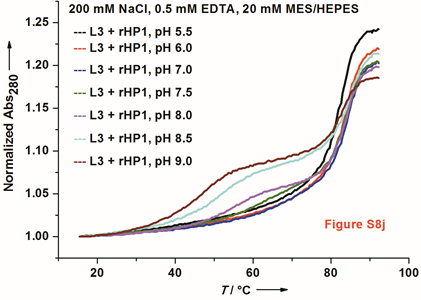 \| \| 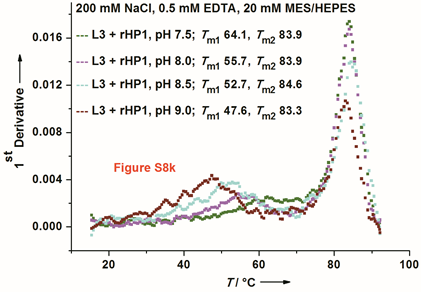 \| 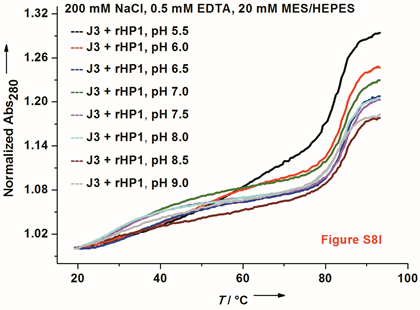 \| \| 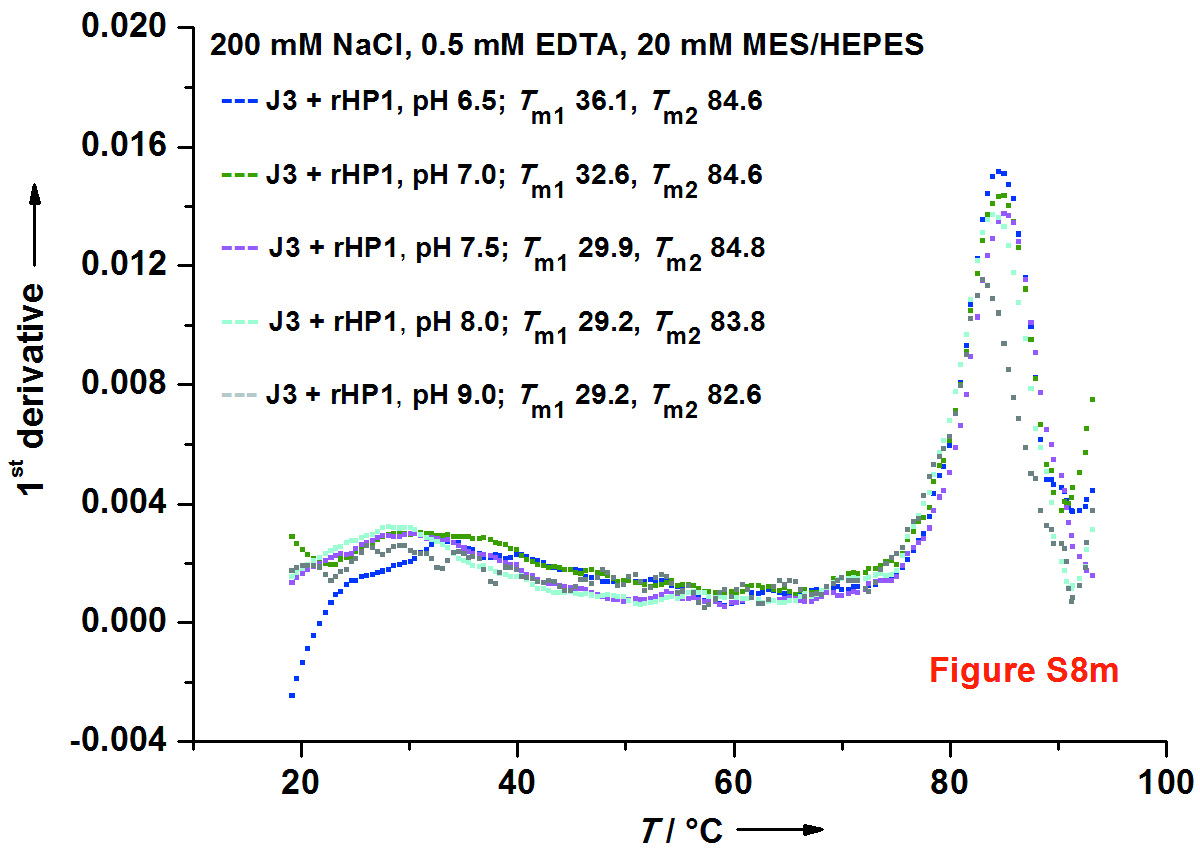 \| 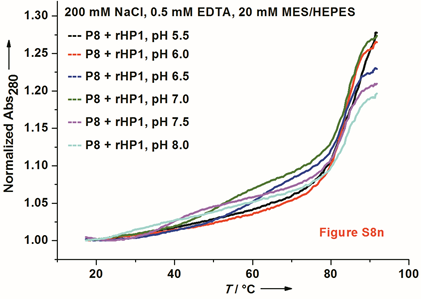 \| \| 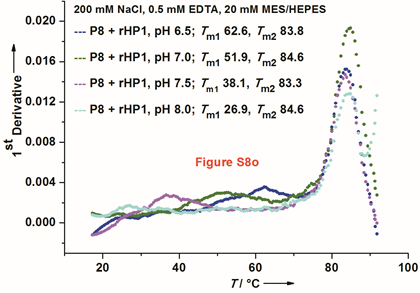 \| 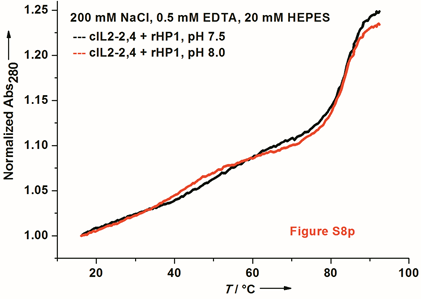 \| \| 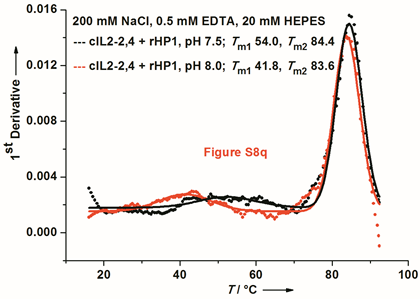 \| 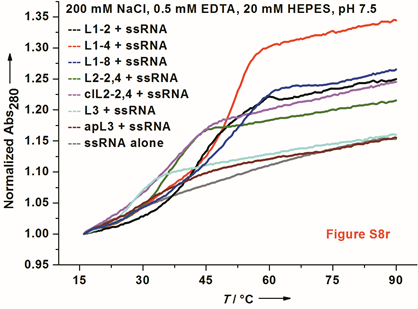 \| \| 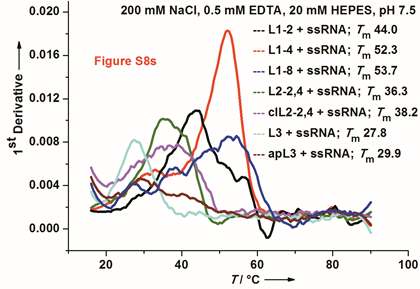 \| 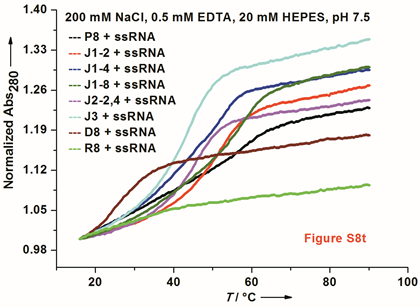 \| \| 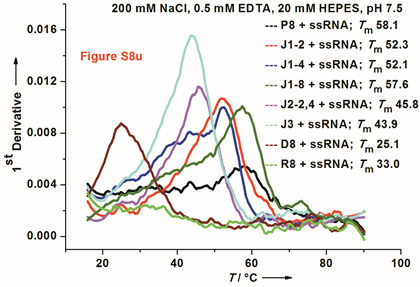 \| 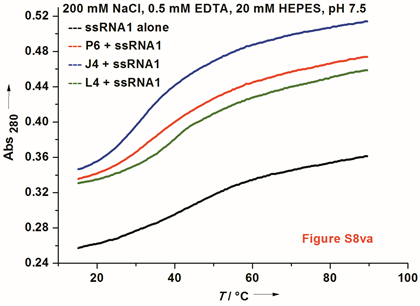 \| \| 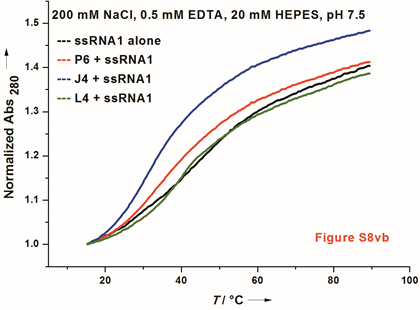 \| 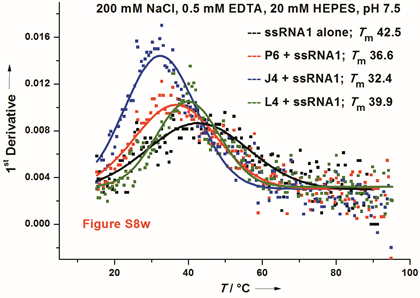 \| \| 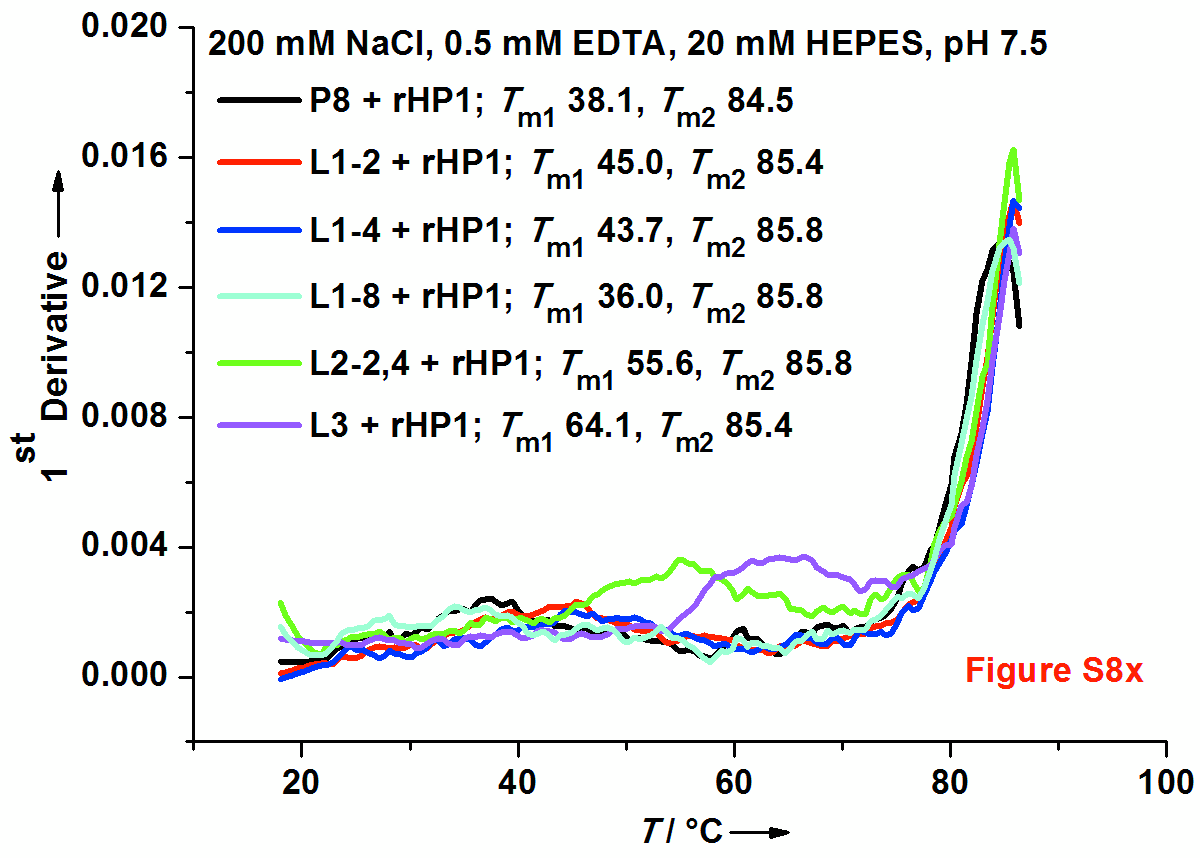 \| 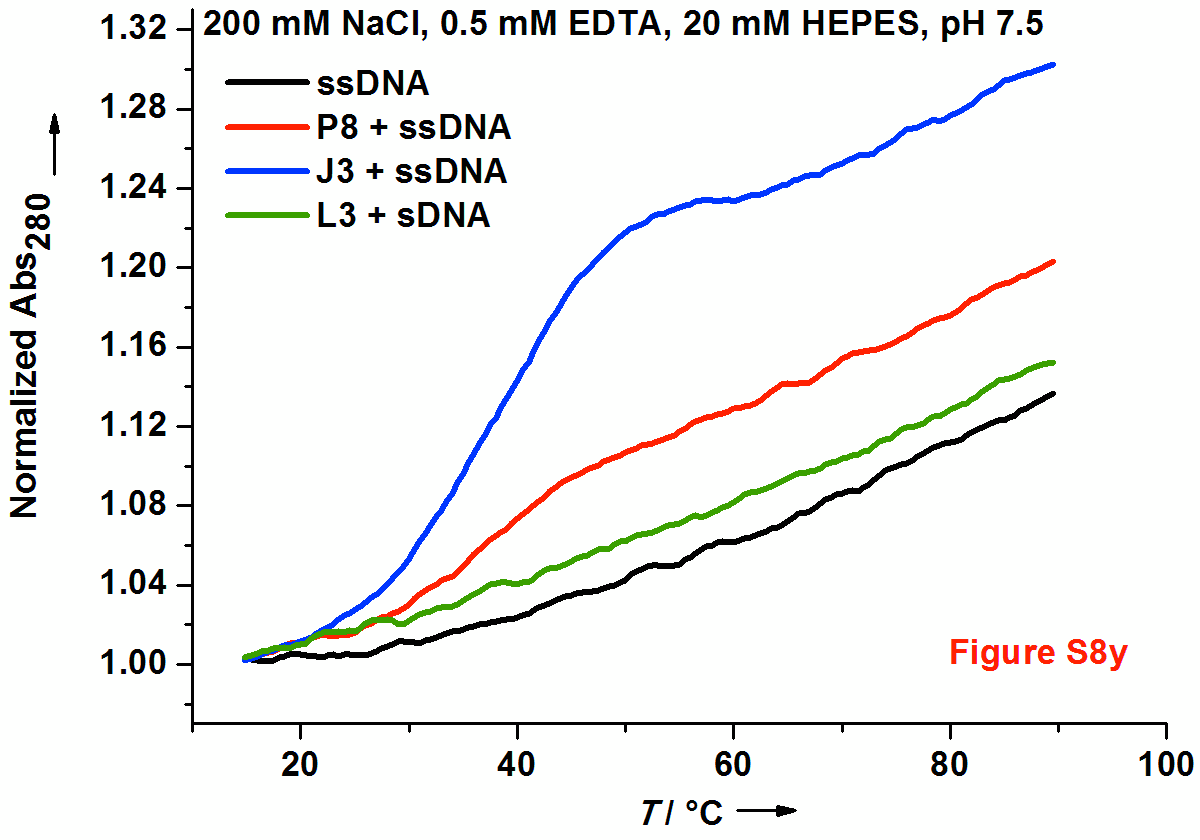 \| \| 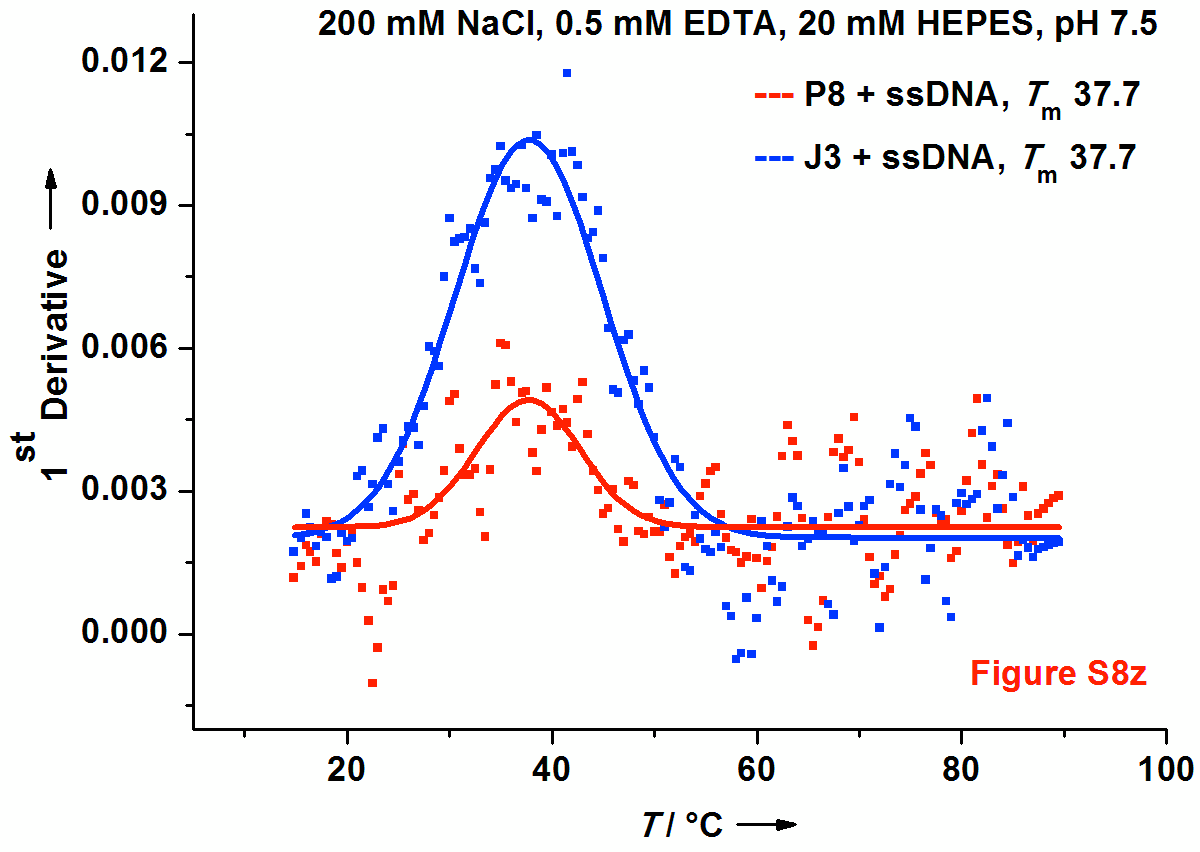 \|  \|   The oligonucleotide concentrations were measured by absorbance at 260 nm in H_2_O at 85 °C, with the extinction coefficients obtained from the MeltWin program. The PNA oligomer concentrations were measured by absorbance at 260 nm in H_2_O at 60 °C, with the extinction coefficients of 8800 M^-1^cm^-1^ for T, and 7300 M^-1^cm^-1^ for C, J, L PNA monomers.  We tested the binding of 6-mer PNAs binding to the 11-mer single-stranded RNA1 (ssRNA1) (5′-GGGAAGGCCAG-3′), which is part of the HIV-HP sequence (see **Figure 2h**).However, the 11-mer ssRNA1 may form a selfcomplementary duplex, including the one shown below.    5′-GGGAAGGCCAG-3′  3′-GACCGGAAGGG-5′  The self-structure of the 11-mer ssRNA1 has a melting transition (see **panel S8v**). Thus, it is not clear, based on the thermal melting results, whether the three 6-mer PNAs bind to the 11-mer single-stranded RNA1 (ssRNA1) (5′-GGGAAGGCCAG-3′). We did not observe clear triplex melting transitions for the 6-mer PNAs binding to HIV-HP, presumably due to low hyperchromicity. |
| --- | --- | --- | --- | --- | --- | --- | --- | --- | --- | --- | --- | --- | --- | --- | --- | --- | --- | --- | --- | --- | --- | --- | --- | --- | --- | --- | --- | --- |

1.
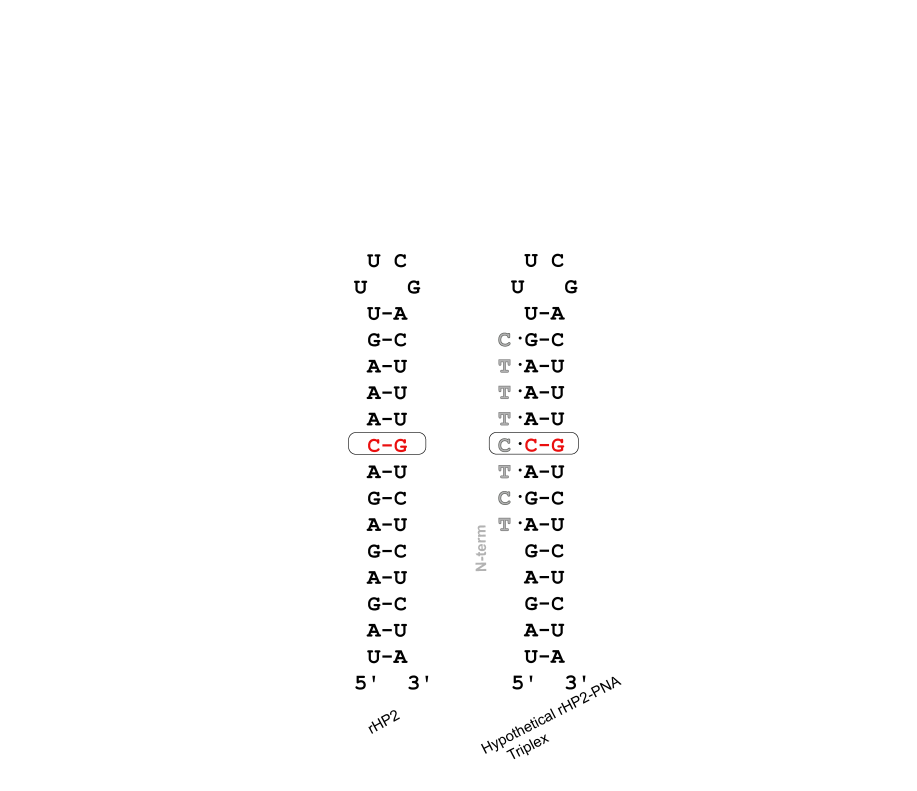
***
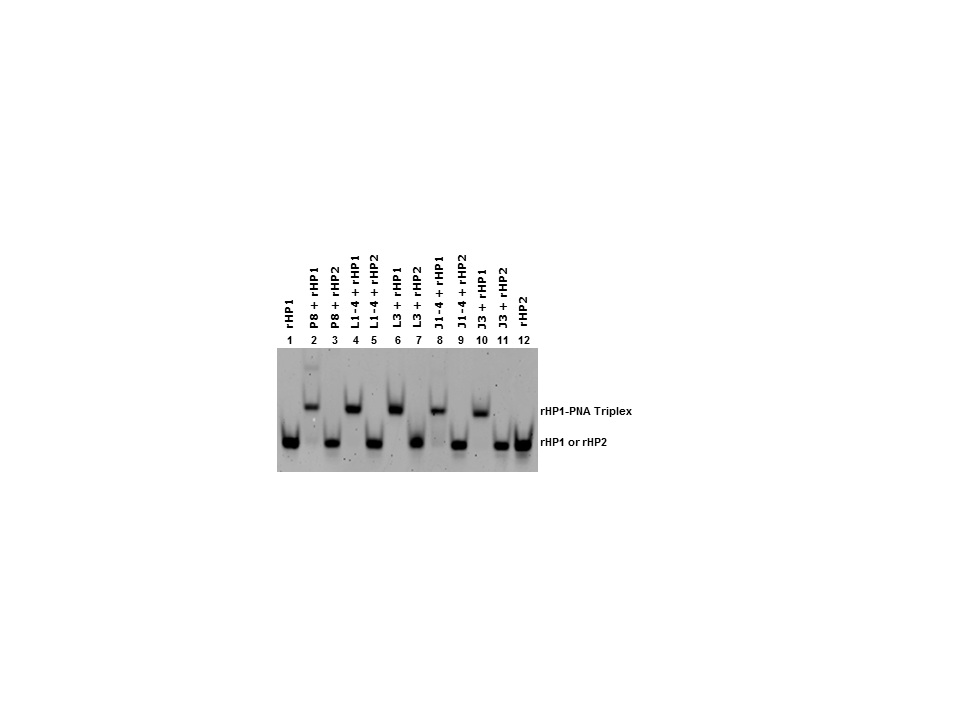
*
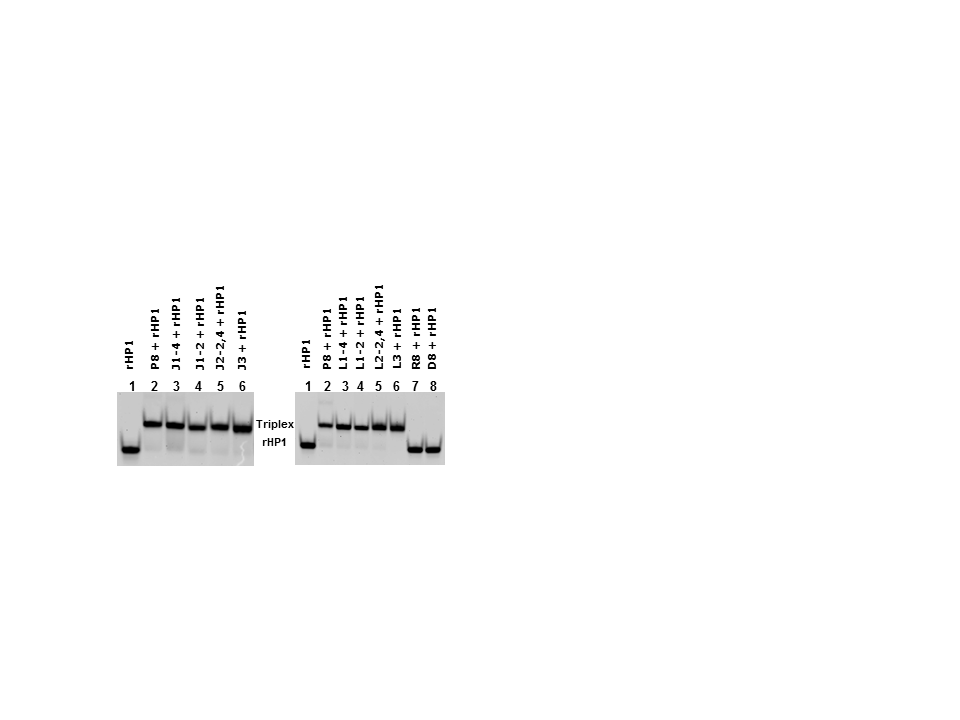
Non-denaturing PAGE results.**

**
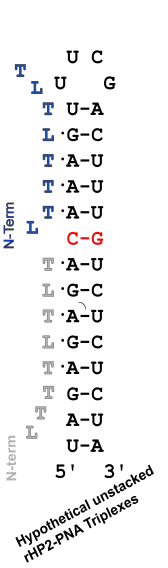
**
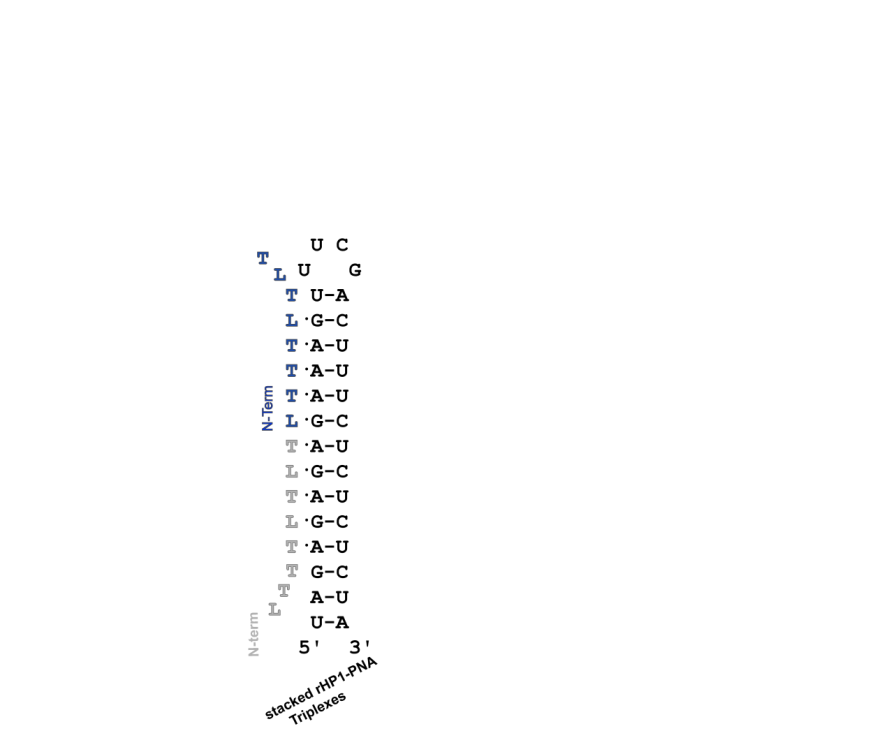


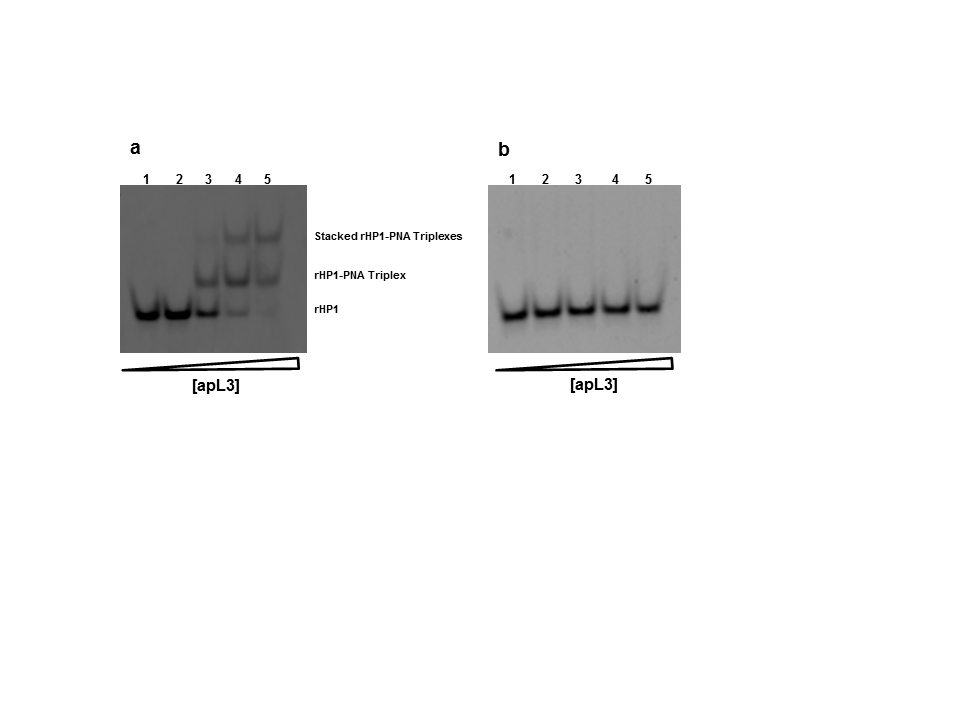


rHP2 + apL3

rHP1 + apL3

**Figure S9.** (**Top**) Non-denaturing PAGE for various PNAs binding to rHP1 and rHP2 in an incubation buffer of 10 mM NaCl, 0.5 mM EDTA, 20 mM MES, pH 5.5. The loaded samples contain 1.3 µM rHP1 with or without 6.5 µM PNA/oligonucleotides in 30 µL. All PNAs form (RNA)_2_-PNA triplex with rHP1, whereas neither oligonucleotide R8 or D8 binds to rHP1. None of the PNAs forms a triplex with rHP2, which has a G-C pair inverted compared to rHP1. (**Bottom**) Non-denaturing PAGE for PNA apL3 binding to rHP1 and rHP2 in an incubation buffer of 200 mM NaCl, 0.5 mM EDTA, 20 mM HEPES (pH 7.5). rHP1 and rHP2 were loaded at 1 µM in 20 µL. The concentrations of PNA apL3 in lanes 1-5 are 0, 1, 5, 10, and 25 µM, respectively. We speculate that, at 4 °C, PNA apL3 may form up to two coaxially-stacked 5-base-triple parallel triplexes (see the structure shown on the right to the gels). Our thermal melting results show no triplex melting transitions for PNA apL3 at pH 5.5-7.5, which suggests the 5-base-triple parallel triplex structures are not stable at >20 °C or hyperchromicity is too low to show a transition. Interestingly, non-denaturing PAGE suggests that apL3 does not bind to rHP2, probably because one base triple and the coaxial stacking are disrupted. Taken together, the results indicate that the first rHP1-apL3 triplex forms at the top part of rHP1 (close to the UUCG tetraloop), and the second triplex does not form without the formation of the first triplex.

***
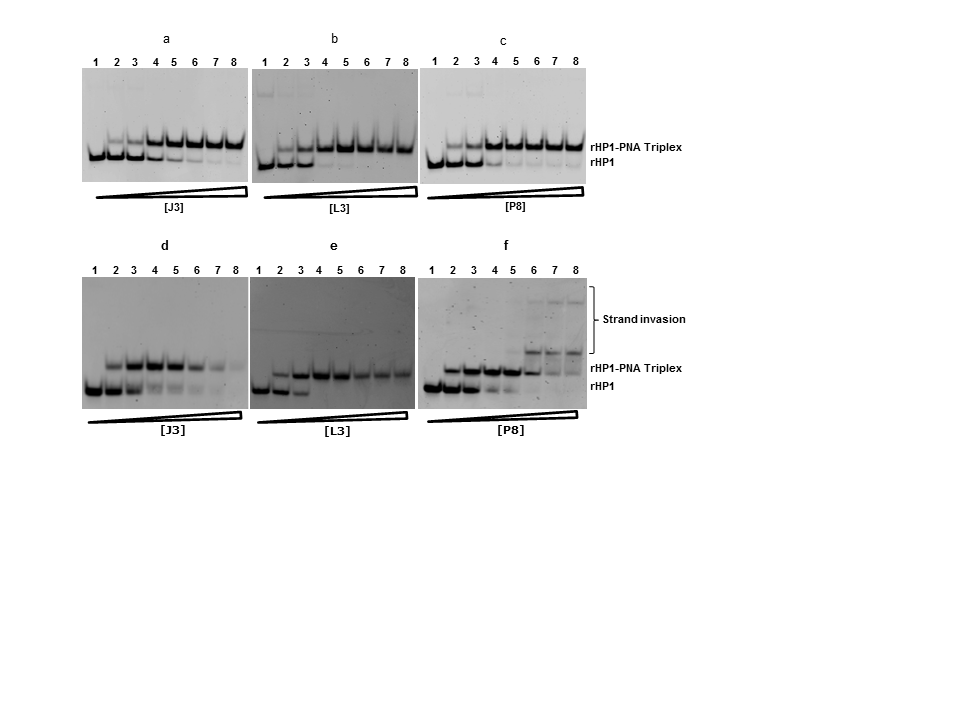
***

**Figure S10**: Strand invasion test for PNAs J3, L3**,** P8 binding to rHP1 in an incubation buffer of 200 mM NaCl, 0.5 mM EDTA, 20 mM HEPES, pH 7.0 **(a-c),** and 10 mM NaCl, 0.5 mM EDTA, 20 mM MES, pH 5.5 (**d-f**). Loaded rHP1 is at 1.3 µM in 30 µL. Molar concentration ratios for rHP1:PNA in lanes 1-8 are 1:0, 1:0.5, 1:1, 1:3, 1:5, 1:10, 1:25, and 1:40, respectively. Strand invasion was observed for P8 at low pH and low salt with PNA:RNA ratio at 10 or greater. Both triplex and hairpin band intensities decrease with increasing concentration of PNA J3 (from lane 6-8, see panel **d**), indicating that aggregation may occur at this condition.

| 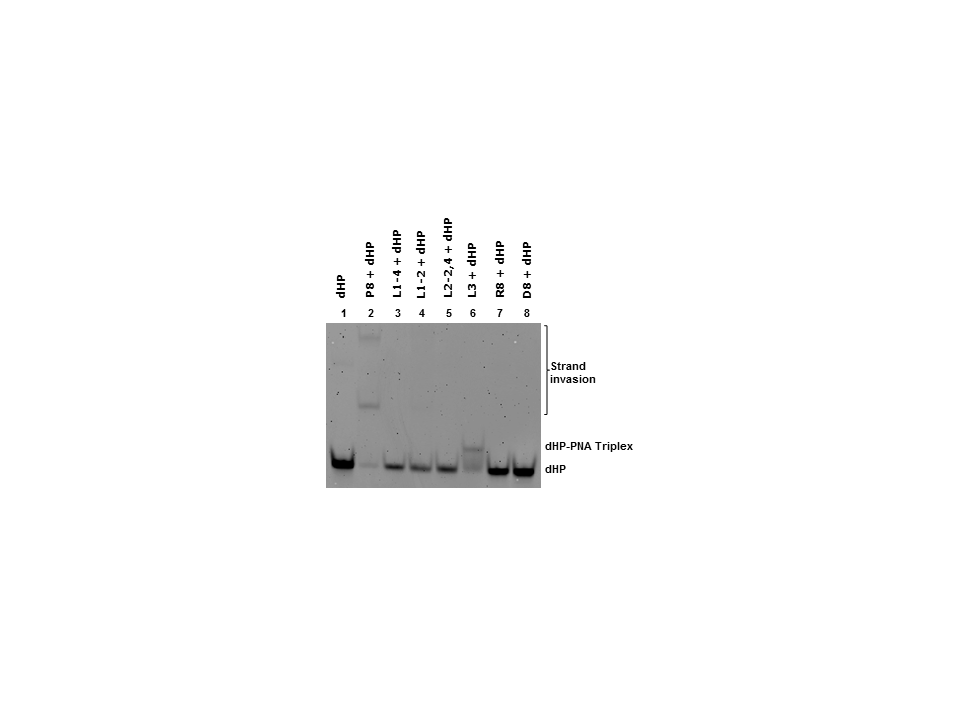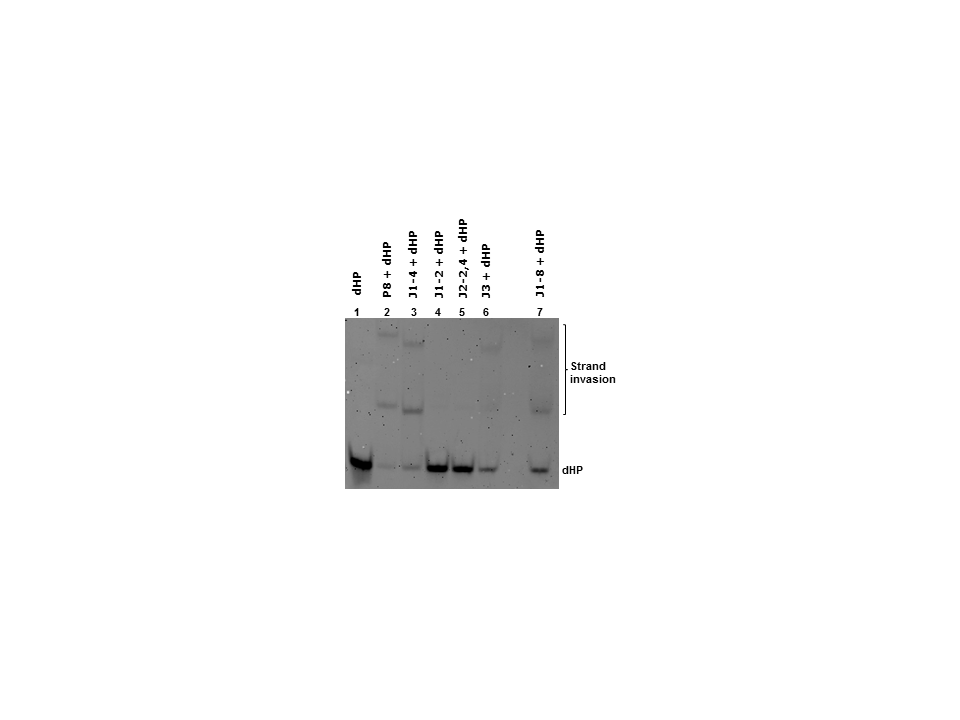 | 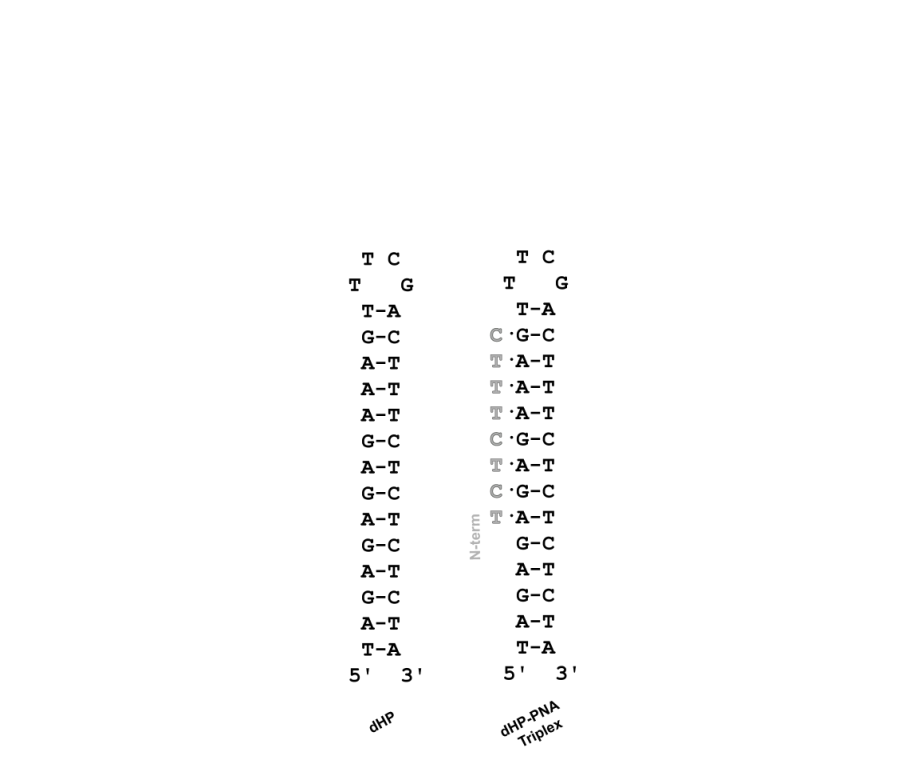 |
| --- | --- |

**Figure S11.** Non-denaturing PAGE for J and L modified PNAs binding to a homologous DNA hairpin of rHP1 (dHP) of rHP1 in an incubation buffer of 10 mM NaCl, 0.5 mM EDTA, 20 mM MES, pH 5.5. The loaded samples contain 1.3 µM dHP with or without 6.5 µM PNA in 30 µL. Only PNA L3 was observed to form (DNA)_2_-PNA triplex at this condition.


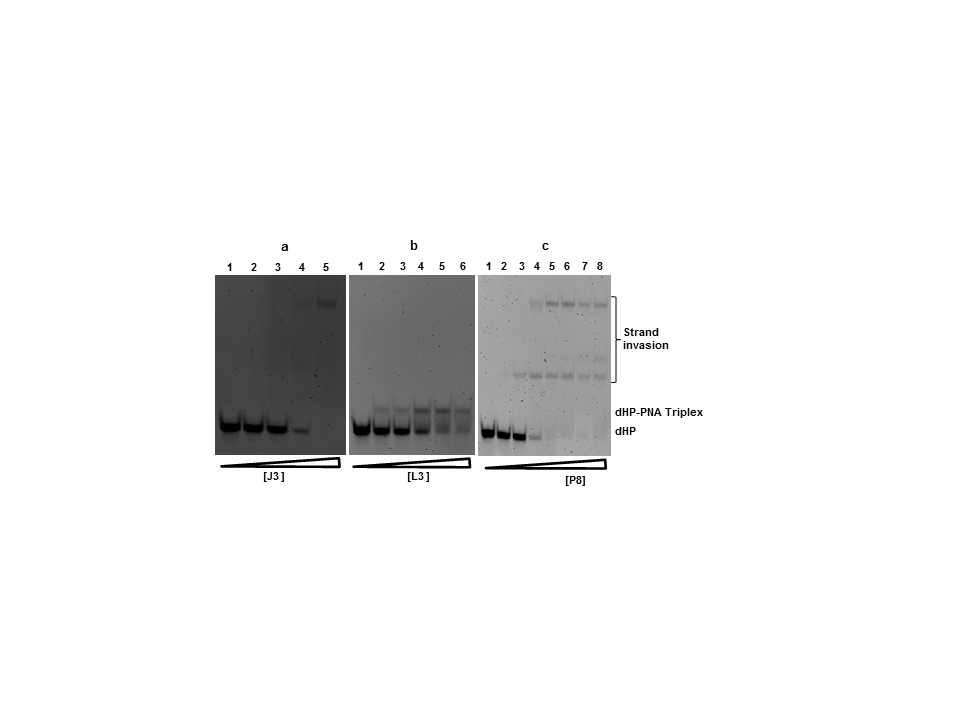


**Figure S12.** Strand invasion test for J3 **(a)**, L3 **(b)** and P8 **(c)** binding to dHP in an incubation buffer of 10 mM NaCl, 0.5 mM EDTA, 20 mM MES, pH 5.5. Loaded dHP is at 1.3 µM in 30 µL. Molar concentration ratios for dHP:PNA in lanes 1-8 are 1:0, 1:0.5, 1:1, 1:3, 1:5, 1:10, 1:25, and 1:40, respectively. Only PNA L3 forms (DNA)_2_-PNA triplex at this condition.


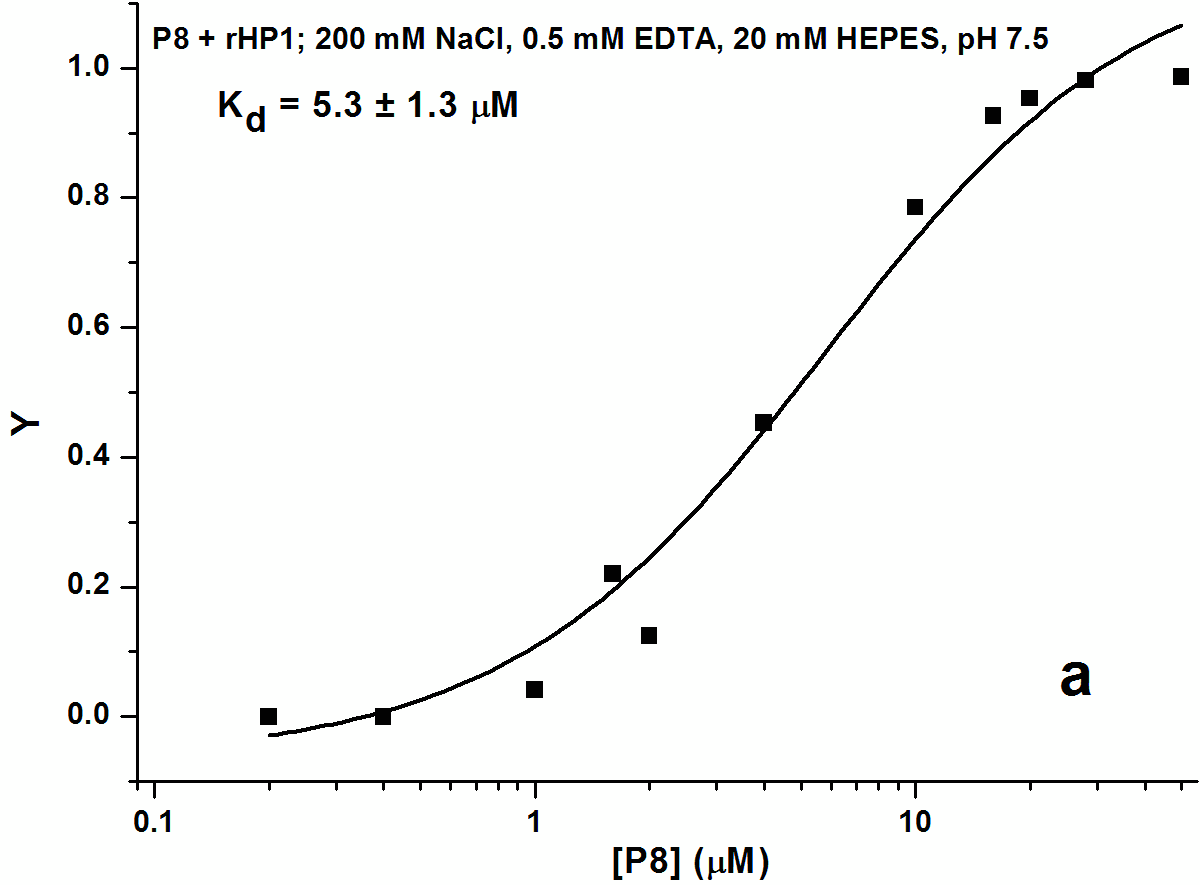

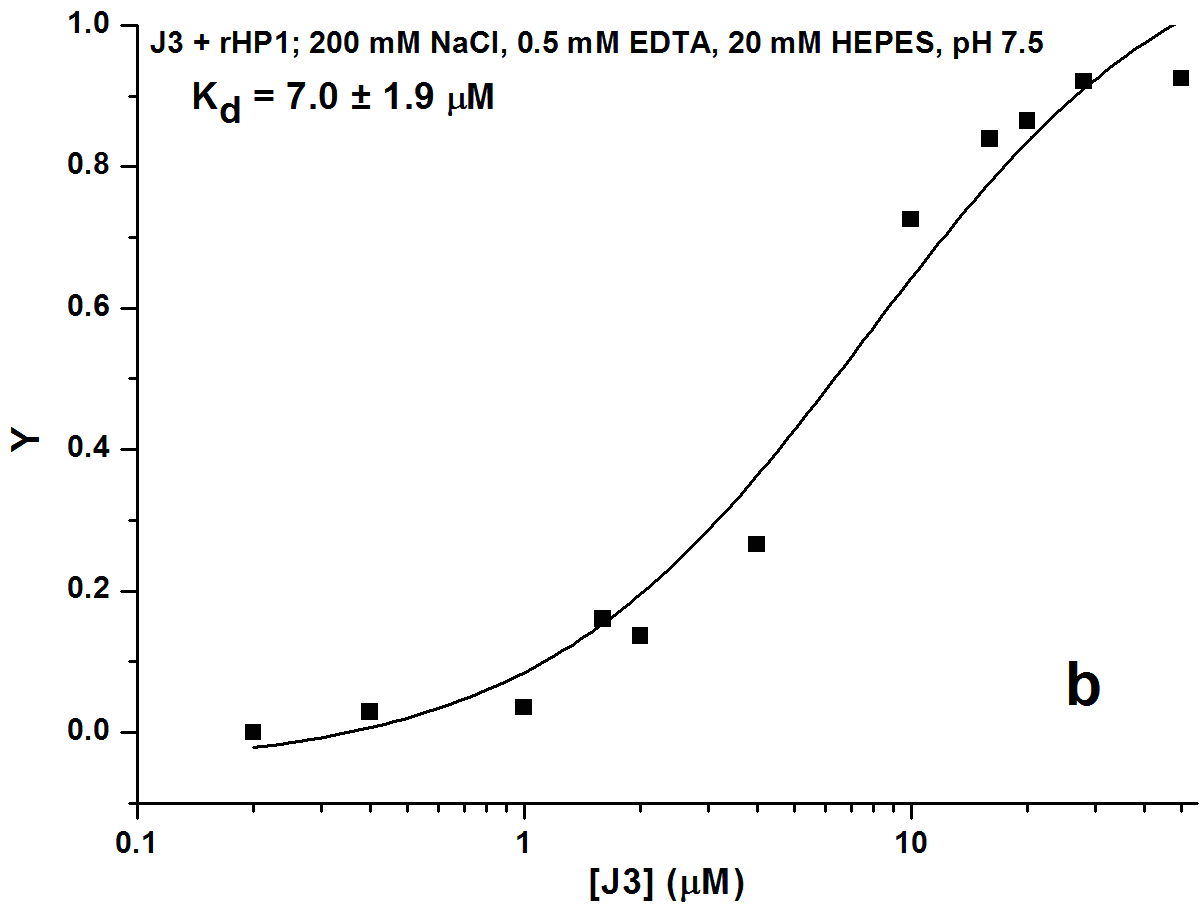

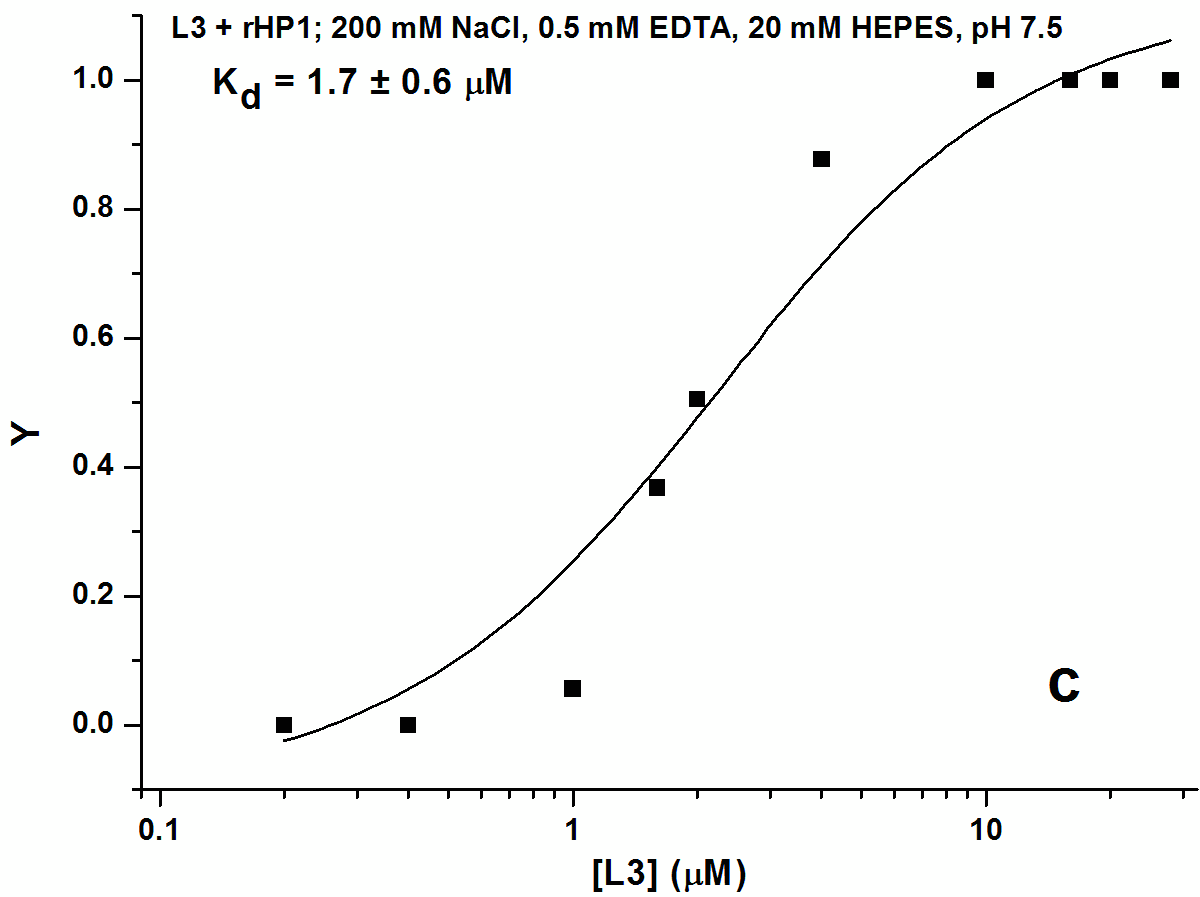

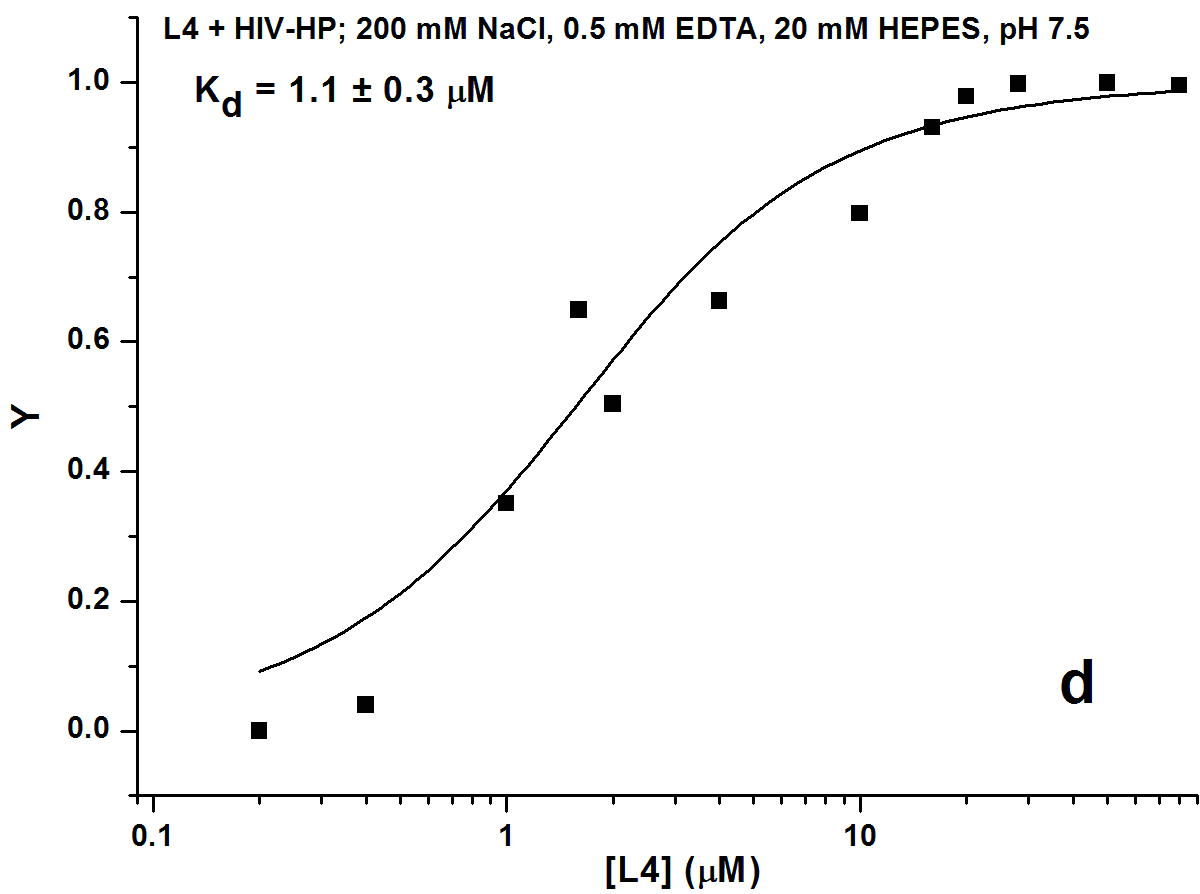


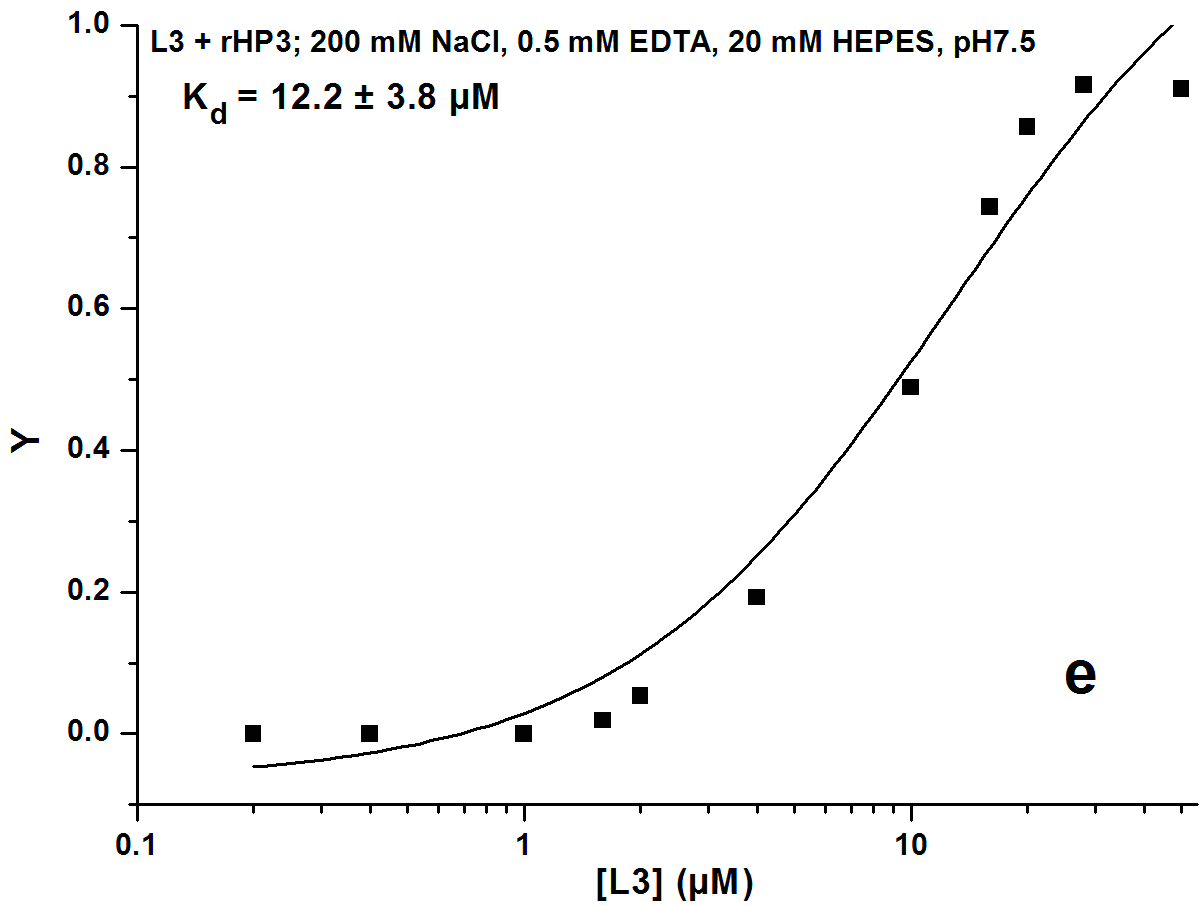

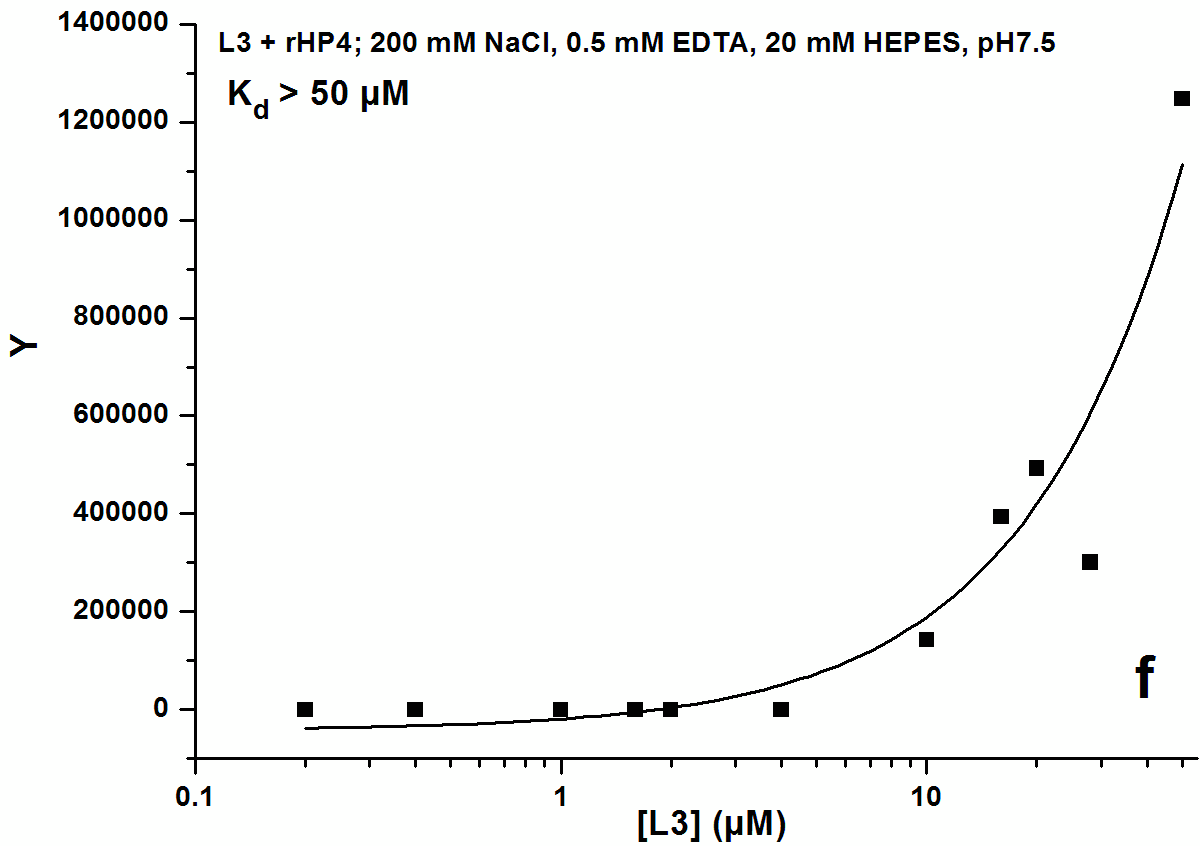


**Figure S13.** *K*_d_ determination by non-denaturing PAGE (see **Figure 4**) for (**a-c**) P8, J3, L3 binding to rHP1, **(d)** L4 binding to HIV-HP, (**e**) L3 binding to rHP3, and **(f)** L3 binding to rHP4 in an incubation buffer of 200 mM NaCl, 0.5 mM EDTA, 20 mM HEPES, pH 7.5. Except for panel (**f**), the fraction of triplex formation in each lane was calculated according to Y = aI_triplex_/(I_duplex_ + aI_triplex_). The band intensities for duplex and triplex are normalized according to a = I_duplex only_/ I_triplex only_. I_duplex only_ is the band intensity for hairpin alone without adding PNA. I_triplex only_ is the band intensity for the lane with the highest concentration of PNA added. The data were then fit to the equation: Y = Y_0_ + B/2R_0_{R_0_ + X + *K*_d_ – [(R_0_ + X + *K*_d_)^2^ – 4R_0_X]^1/2^} where R_0_ is the total RNA hairpin concentration (1 µM). Y_0_ is the minimum fraction of triplex formation. B is the maximum fraction of triplex formation. X is total PNA concentration. *K*_d_ is the dissociation constant. For panel **f**, only triplex bands were used for *K*_d_ approximation.


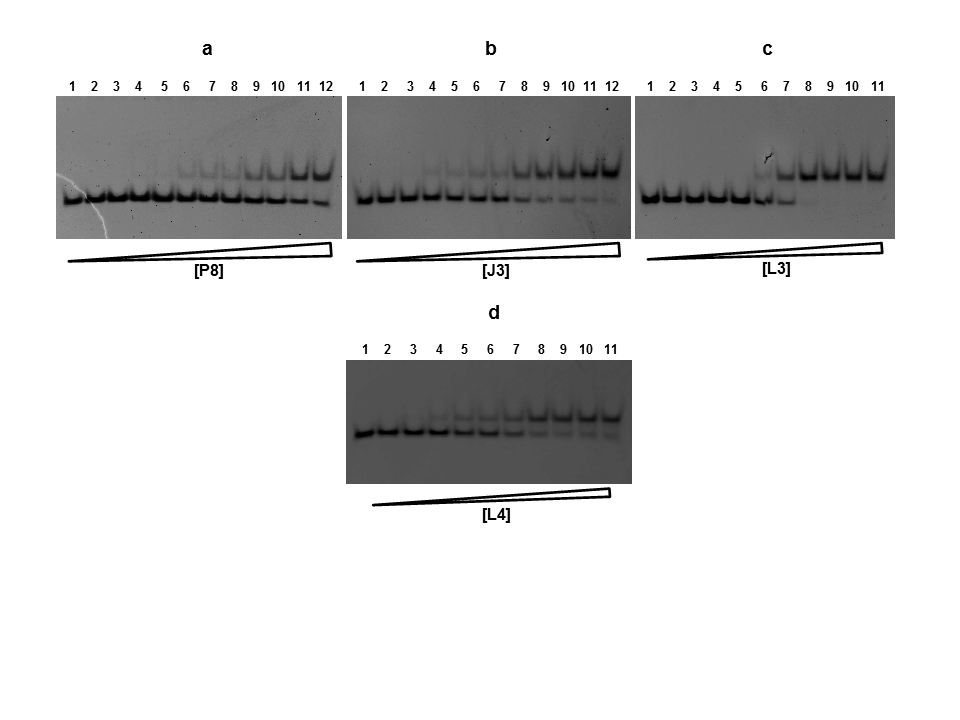


**Figure S14.** Non-denaturing PAGE for **(a-c)** P8, J3, L3 binding to rHP1 and **(d)** L4 binding to HIV-HP in an incubation buffer 200 mM NaCl, 0.5 mM EDTA, 20 mM HEPES, pH 8.0. Loaded rHP1 and HIV-HP are at 1 µM in 20 µL. Concentrations for PNA in lanes 1-12 are: 0, 0.2, 0.4, 1, 1.6, 2, 4, 10, 16, 20, 28, and 50 µM, respectively.


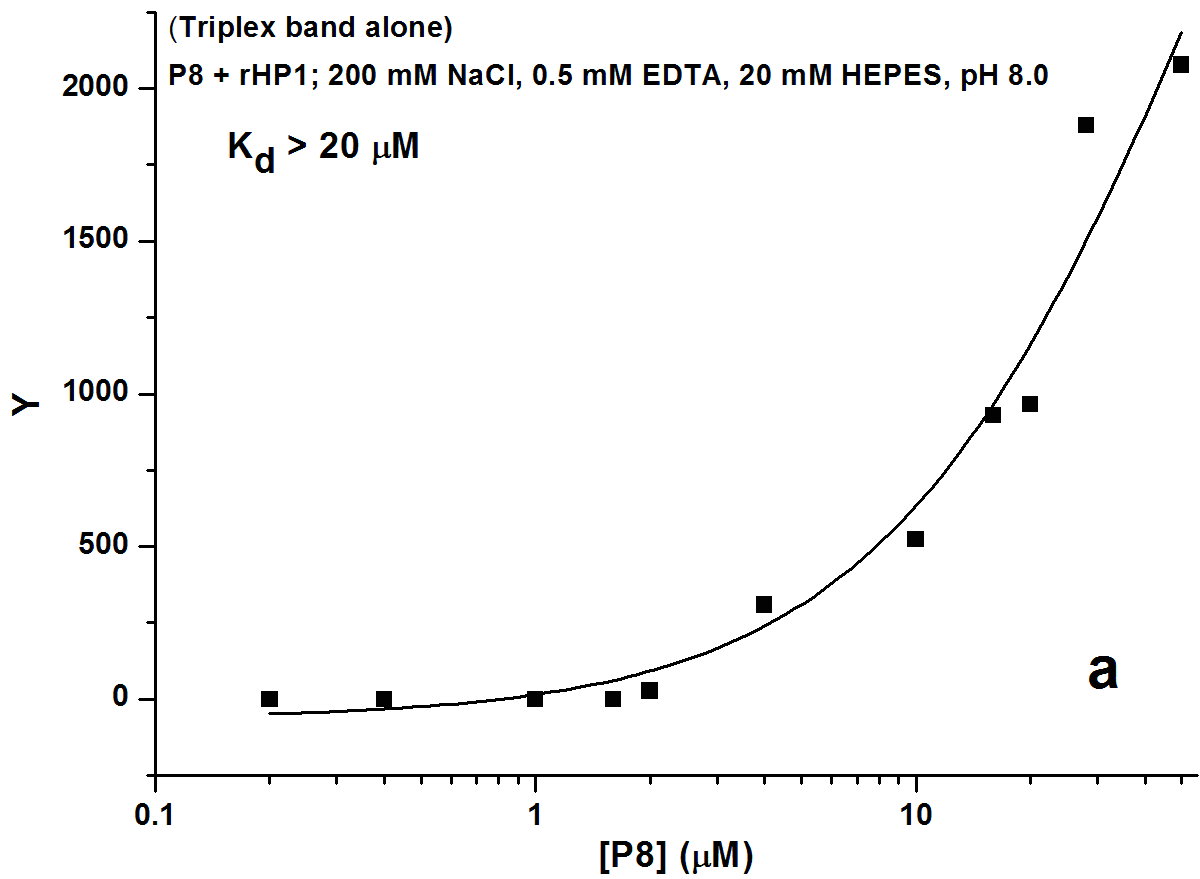

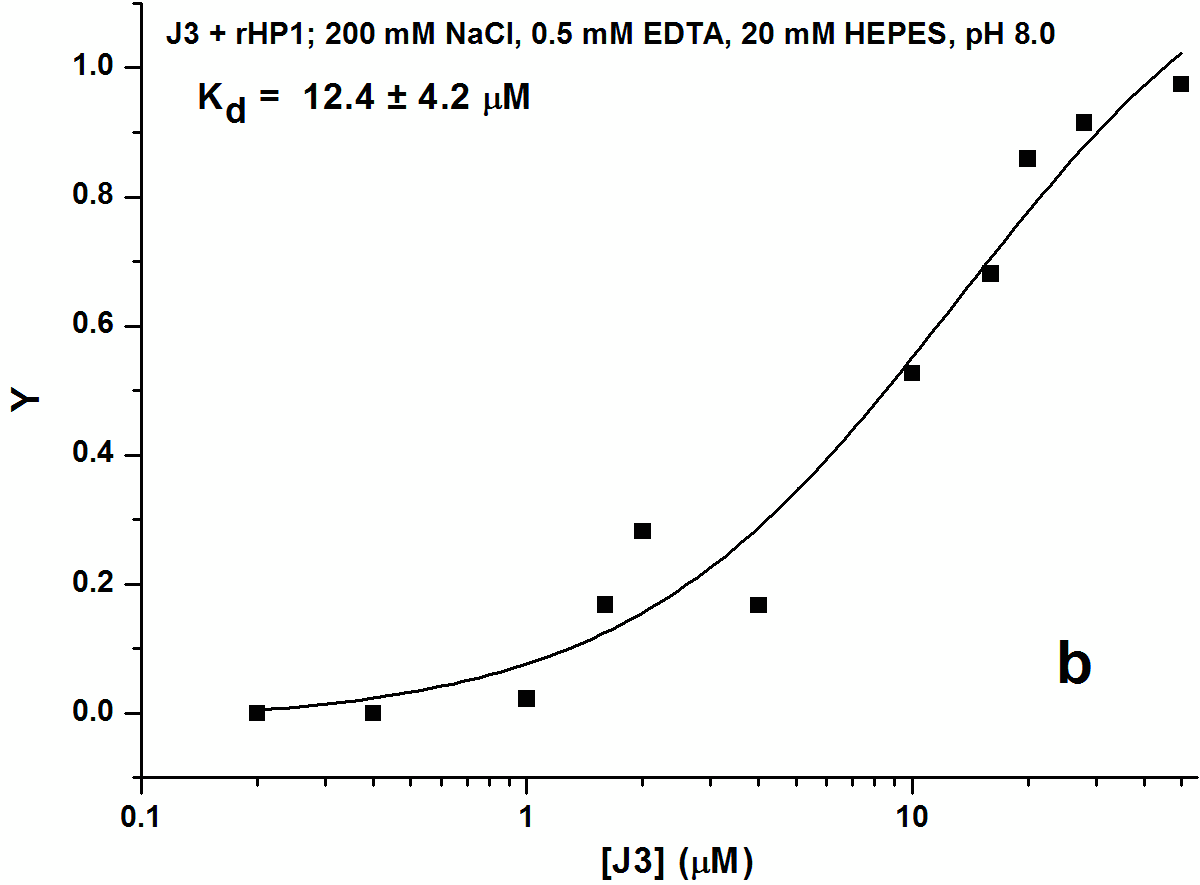

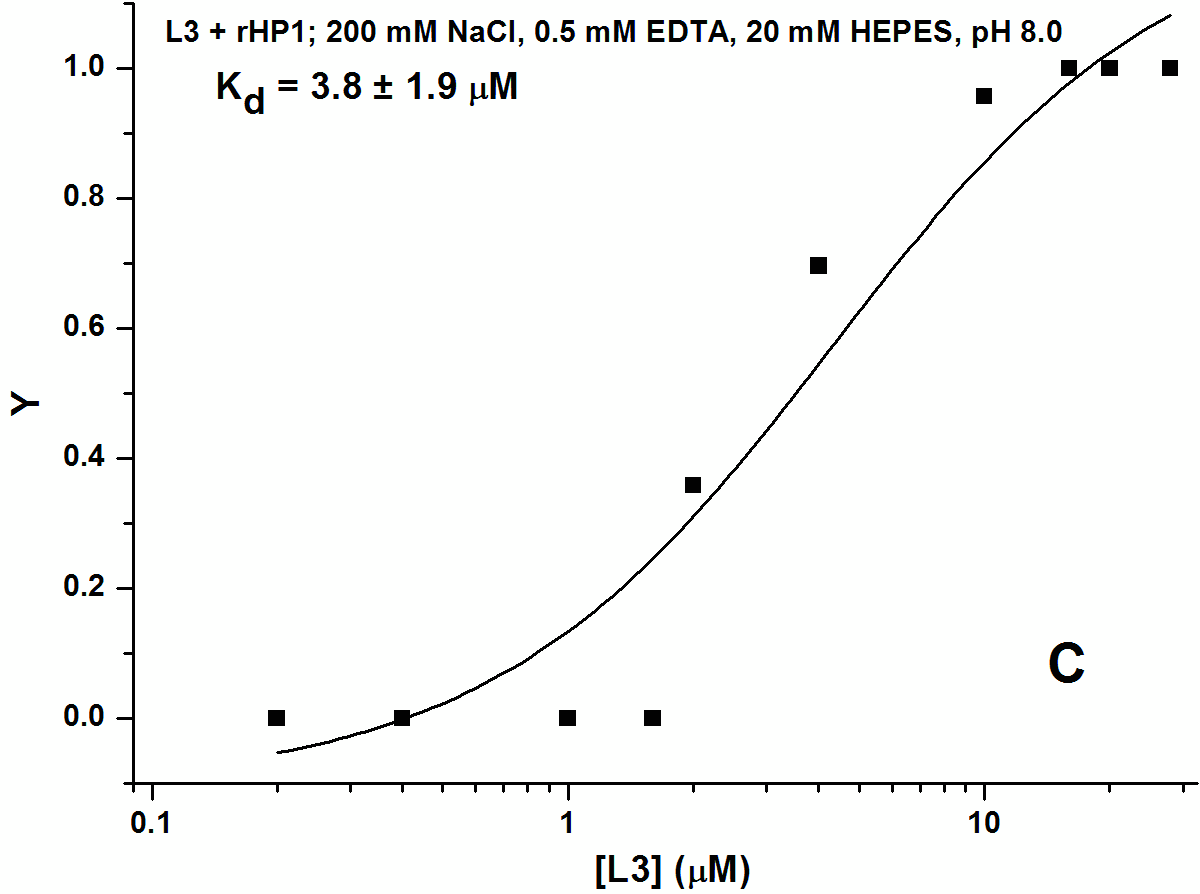

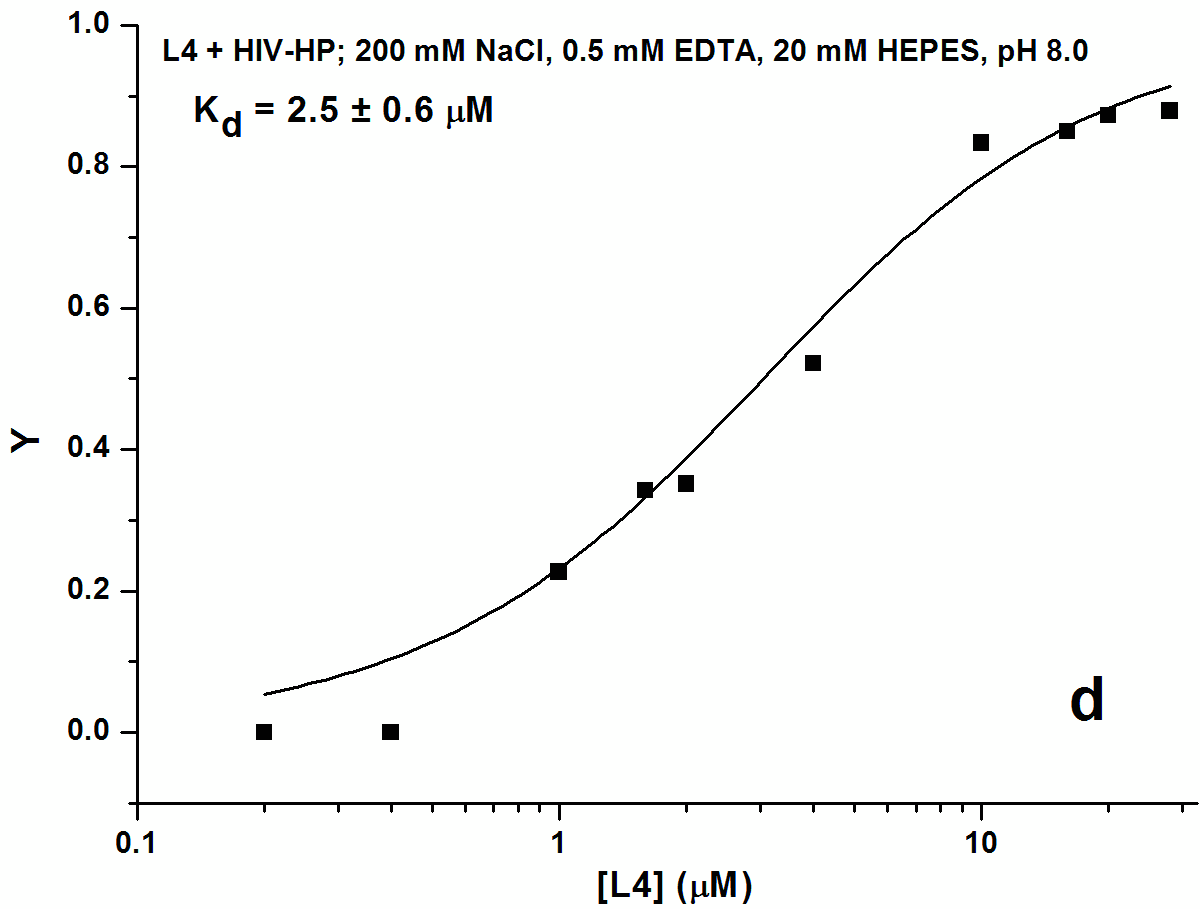


**Figure S15.** *K*_d_ determination by non-denaturing PAGE (**Figure S14**) for (**a-c**) P8, J3, L3 binding to rHP1 and **(d)** L4 binding to HIV-HP in an incubation buffer 200 mM NaCl, 0.5 mM EDTA, 20 mM HEPES, pH 8.0. For panel **a**, only triplex bands were used for *K*_d_ approximation.


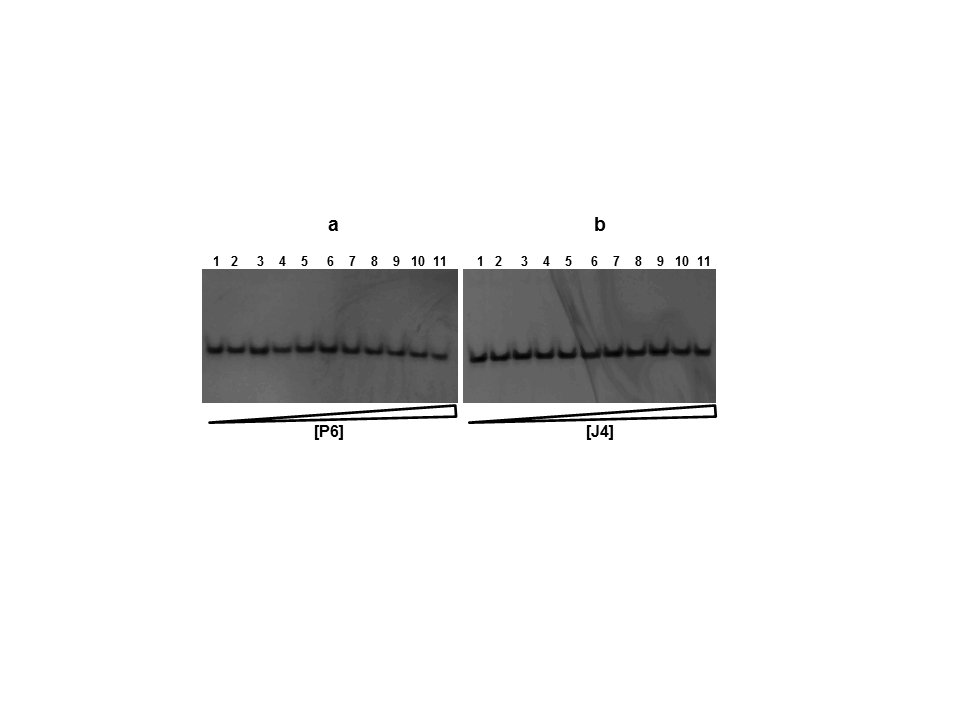


**Figure S16.** Non-denaturing PAGE for **(a)** P6 and **(b)** J4 binding to HIV-HP in an incubation buffer 200 mM NaCl, 0.5 mM EDTA, 20 mM HEPES, pH 7.0. HIV-HP was loaded at 1 µM in 20 µL. Concentrations for PNA in lanes 1-11 are 0, 0.2, 0.4, 1, 1.6, 2, 4, 10, 16, 20, and 28 µM, respectively. No binding was observed at this condition.

1. **Computational details.**

DFT calculations were carried out with the Gaussian 09 package (65). The bases geometries are fully optimized by B3LYP (66-68) method with 6-31G+(d,p) basis set. Ultrafine integration grids are used in the calculation with tight convergence criteria. Harmonic frequency calculations are carried out to calculate the free energy in 298.15 K.

**Table S2.** The proton and bases’ free energy in the gas phase at 298.15 K.

| Compound | Δ*G*° in 298.15 K (a.u.) |
| --- | --- |
| H^+^ | -0.0100 |
| C | -434.1823 |
| *N*^3^ protonated C cation | -434.5496 |
| 2-Thio C | -757.1329 |
| *N*^3^ protonated 2-thio C cation | -757.4989 |
| J | -434.1925 |
| *N*^3^ deprotonated J anion | -433.6530 |
| L | -757.1495 |
| *N*^3^ deprotonated L anion | -756.6213 |
| T | -493.3746 |
| *N*^3^ deprotonated T anion | -492.8228 |
| 2-Thio T | -816.3297 |
| *N*^3^ deprotonated 2-thio T anion | -815.7836 |
| A | -506.5634 |
| *N*^7^ protonated A cation | -506.9146 |
| G | -581.7972 |
| *N*^7^ protonated G cation | -582.1672 |

**Table S3.** The free energy change ΔΔ*G* for the deprotonation process in the gas phase.

| Deprotonation process | ΔΔ*G* at 298.15 K (kJ/mol) | p*K*_a_^*^ |
| --- | --- | --- |
| CH^+^ → C + H^+^ | 938.09 | 164.33 |
| 2-thio C^+^ → 2-thio C + H^+^ | 934.68 | 163.73 |
| J → J^-^ + H^+^ | 1390.20 | 243.52 |
| L → L^-^ + H^+^ | 1360.53 | 238.32 |
| T → T^-^ + H^+^ | 1422.50 | 249.18 |
| 2-thio T → 2-thio T^-^ + H^+^ | 1407.53 | 246.56 |
| A^+^ → A + H^+^ | 1030.71 | 180.55 |
| G^+^ → G + H^+^ | 945.18 | 165.57 |

^*^$\Delta G^{\emptyset}=2.303RTpK_{a}$

1. References.

34. Egholm, M., Christensen, L., Dueholm, K.L., Buchardt, O., Coull, J. and Nielsen, P.E. (1995) Efficient pH-independent sequence-specific DNA-binding by pseudoisocytosine-containing bis-PNA. *Nucleic Acids Res.*, **23**, 217-222.

46. Fissekis, J.D. and Sweet, F. (1970) Synthesis of 5-carboxymethyluridine. A nucleoside from transfer ribonucleic acid. *Biochemistry*, **9**, 3136-3142.

65. Gaussian 09, Revision **A.1**, Frisch, M.J., Trucks, G.W., Schlegel, H.B., Scuseria, G. E., Robb, M.A., Cheeseman, J.R., Scalmani, G., Barone, V., Mennucci, B., Petersson, G.A., Nakatsuji, H., Caricato, M., Li, X., Hratchian, H. P., Izmaylov, A.F., Bloino, J., Zheng, G., Sonnenberg, J.L., Hada, M., Ehara, M., Toyota, K., Fukuda, R., Hasegawa, J., Ishida, M., Nakajima, T., Honda, Y., Kitao, O., Nakai, H., Vreven, T., Montgomery, Jr., J.A., Peralta, J.E., Ogliaro, F., Bearpark, M., Heyd, J.J., Brothers, E., Kudin, K.N., Staroverov, V.N., Kobayashi, R., Normand, J., Raghavachari, K., Rendell, A., Burant, J.C., Iyengar, S.S., Tomasi, J., Cossi, M., Rega, N., Millam, N.J., Klene, M., Knox, J.E., Cross, J.B., Bakken, V., Adamo, C., Jaramillo, J., Gomperts, R., Stratmann, R.E., Yazyev, O., Austin, A.J., Cammi, R., Pomelli, C., Ochterski, J.W., Martin, R. L., Morokuma, K., Zakrzewski, V.G., Voth, G.A., Salvador, P., Dannenberg, J.J., Dapprich, S., Daniels, A.D., Farkas, Ö., Foresman, J.B., Ortiz, J.V., Cioslowski, J., Fox, D.J. (2009) Gaussian, Inc., Wallingford CT.

66. Becke, A.D. (1993) A New Mixing of Hartree-Fock and Local Density-Functional Theories. *J. Chem. Phys.*, **98**, 1372-1377.

67. Becke, A.D. (1993) Density-Functional Thermochemistry .III. The Role of Exact Exchange. *J. Chem. Phys.*, **98**, 5648-5652.

68. Lee, C., Yang, W., Parr, R.G. (1988) Development of the Colle-Salvetti Correlation-Energy Formula into a Functional of the Electron-Density. *Phys. Rev. B*, **37**, 785-789.
